# A Randomized Clinical Trial to Evaluate Two Doses of an Intra-Articular Injection of LMWF-5A in Adults with Pain Due to Osteoarthritis of the Knee

**DOI:** 10.1371/journal.pone.0087910

**Published:** 2014-02-03

**Authors:** David Bar-Or, Kristin M. Salottolo, Holli Loose, Matthew J. Phillips, Brian McGrath, Nathan Wei, James L. Borders, John E. Ervin, Alan Kivitz, Mark Hermann, Tammi Shlotzhauer, Melvin Churchill, Donald Slappey, Vaughan Clift

**Affiliations:** 1 Trauma Research Department, Swedish Medical Center, Englewood, Colorado, United States of America; 2 Trauma Research Department, St Anthony Hospital, Lakewood, Colorado, United States of America; 3 Ampio Pharmaceuticals, Inc., Greenwood Village, Colorado, United States of America; 4 Rocky Vista University, Aurora Colorado, United States of America; 5 University Orthopaedic Center, Amherst, New York, United States of America; 6 Arthritis Treatment Center, Frederick Maryland, United States of America; 7 Central Kentucky Research Associates, Lexington, Kentucky, United States of America; 8 Center for Pharmaceutical Research, Kansas City, Missouri, United States of America; 9 Altoona Center for Clinical Research, Duncansville, Pennsylvania, United States of America; 10 Danville Orthopeadic Clinic, Danville Virginia, United States of America; 11 Rochester Medical Research, Rochester, New York, United States of America; 12 Physician Research Collaboration, Lincoln Nebraska, United States of America; 13 Alabama Clinical Therapeutics, LLC, Birmingham, Alabama, United States of America; Center for Rheumatic Diseases, India

## Abstract

**Objective:**

The Low Molecular Weight Fraction of 5% human serum Albumin (LMWF-5A) is being investigated as a treatment for knee pain from osteoarthritis.

**Methods:**

This was a multicenter randomized, vehicle-controlled, double-blind, parallel study designed to evaluate the safety and efficacy of two doses of an intra-articular injection of LMWF-5A. Patients with symptomatic knee osteoarthritis were randomized 1∶1∶1∶1 to receive a single 4 mL or 10 mL intra-articular knee injection of either LMWF-5A or vehicle control (saline). The primary efficacy endpoint was the difference between treatment groups in the Western Ontario and McMaster Universities (WOMAC) pain change from baseline over 12 weeks. Safety was examined as the incidence and severity of adverse events (AEs).

**Results:**

A total of 329 patients were randomized and received treatment. LMWF-5A resulted in a significant decrease in pain at 12 weeks compared to vehicle control (−0.93 vs −0.72; estimated difference from control: −0.25, p = 0.004); an injection volume effect was not observed (p = 0.64). The effect of LMWF-5A on pain was even more pronounced in patients with severe knee OA (Kellgren Lawrence Grade IV): the estimated difference from control was −0.42 (p = 0.02). Adverse events were generally mild and were similar in patients who received vehicle control (47%) and LMWF-5A (41%).

**Conclusions:**

This clinical trial demonstrated that LMWF-5A is safe and effective at providing relief for the pain of moderate to severe OA of the knee over 12 weeks when administered by intra-articular injection into the knee.

**Trial Registration:**

ClinicalTrials.gov NCT01839331

## Introduction

Osteoarthritis (OA) is the most common form of arthritis affecting a conservatively estimated 27 million Americans in 2008 [1]. Symptomatic OA of the knee occurs in approximately 12% of individuals over the age of 60 [2]. OA is caused by inflammation of the soft tissue and bony structures of the joint which worsens over time and leads to progressive thinning of articular cartilage, narrowing of the joint space, synovial membrane thickening, osteophyte formation and increased density of subchondral bone. These changes eventually result in chronic pain and disability, and despite drug therapy may eventually require surgery for total joint replacement [3].

Current drug treatment for OA of the knee relies on pain control with analgesics, anti-inflammatory treatment with non-steroidal anti-inflammatory drugs (NSAIDs), intra-articular (IA) corticosteroids, and IA hyaluronan products. The only evidence based treatment recommendations by the American Academy of Orthopaedic Surgeons (AAOS) for pain due to OA are self-management/physical activity, weight loss, and NSAIDs or Tramadol; patients with pain that is not controlled by these recommended treatments rely on non-recommended alternatives, or eventually knee replacement [4]. Therefore, there is a need for additional anti-inflammatory and analgesic treatments for OA, particularly as the population ages and the prevalence of obesity, a contributing factor to the development of OA, continues to rise [2], [5], [6], [7].

Human Serum Albumin (HSA) has been commercially approved and in use for over 30 years. It has been safely administered intravenously to humans worldwide and has an excellent safety profile. In common clinical use, HSA is administered as a 5% solution in volumes of 100–500 mL per day in treatment for shock, burns, and plasma volume expansion [8], [9], [10].

The Low Molecular Weight Fraction of 5% HSA (LMWF-5A) has not previously been used for the indication of OA. The low molecular weight fraction (< 5,000 Da) of pharmaceutical HSA contains aspartyl-alanyl diketopiperazine (DA-DKP), which is formed after the dipeptide aspartate-alanine is cleaved from the N-terminus of albumin and cyclizes into a diketopiperazine [8], [11], [12], [13], [14]. DA-DKP has been shown to have multiple anti-inflammatory and immune modulating effects [12], [13], [15], and is believed to be one of the active ingredients in the pharmacological effects of commercial HAS.

LMWF-5A is being developed to provide relief for the pain of moderate to severe OA of the knee. A previous randomized, placebo-controlled, double-blind study conducted in 43 adults in Australia demonstrated that a single 4 mL IA injection of LMWF-5A is considered safe and well tolerated, and is efficacious at reducing pain in adults with OA of the knee (unpublished).

The purpose of this study was to investigate the safety and efficacy of two doses of a single IA knee injection of LMWF-5A on joint pain in OA of the knee. The primary trial objective was to evaluate the greater efficacy of 10 mL LMWF-5A versus 10 mL vehicle control than 4 mL LMWF-5A versus 4 mL vehicle control IA injection in improving knee pain, when applied to patients suffering from OA of the knee. The secondary trial objectives included: the safety of an IA injection of LMWF-5A, and the efficacy of an IA of LMWF-5A on stiffness, function, and overall disease severity.

## Methods

### Ethics statement

The study was performed in accordance with the principles of good clinical practice guidelines and received institutional review board (IRB) approval from the SUNY-Buffalo Health Sciences IRB and Liberty IRB; written informed consent was obtained from all participants involved in the study. Registration on ClinicalTrials.gov was initiated on March 25, 2013 and preceded patient recruitment (Identifier: NCT01839331).

### Study design

We conducted a randomized, vehicle-controlled, double-blind, parallel study designed to evaluate the effect of two doses of a single IA knee injection of LMWF-5A in patients with symptomatic knee OA. The study was conducted at nine clinics across the United States, and consisted of a 28 day screening period and a 12 week participation period. Patients were enrolled and received treatment between March 29, 2013 and May 1, 2013, with follow-up through July 17, 2013.

### Patient selection

Patients were recruited from the population being seen by Investigators at the clinics participating in the study. In addition, we recruited patients through notifications sent to referring physicians as well as radio and web-based campaigns.

Eligible patients were between the ages of 40 to 85 years old, fully ambulatory, with symptomatic index knee OA of at least 6 months preceding screening and a clinical diagnosis of OA supported by recent radiologic evidence within 6 months of screening, with moderate-to-severe OA pain in the index knee (baseline pain rating of ≥ 1.5 on the Western Ontario and McMaster Universities Arthritis Index (WOMAC®) osteoarthritis Index 3.1 5-point Likert pain subscale [16] without evidence of analgesia use 12 hours preceding screening/baseline efficacy measures, and no clinically significant liver abnormality.

Exclusion criteria included: A history of allergic reactions to albumin and its excipients; any human albumin treatment in the 3 months before randomization; concurrent arthritic conditions such as inflammatory or crystal arthropathies, previous major injury, and any other disease or condition interfering with the free use and evaluation of the index knee for the duration of the trial; any pharmacological or non-pharmacological treatment targeting OA started or changed during the 4 weeks prior to randomization; use of the following medications during the study: IA-injected pain medication or topical treatment in the index knee, analgesics containing opioids, significant anticoagulant therapy, immunosuppressants, systemic treatments, or corticosteroids > 10 mg prednisolone equivalent per day.

### Randomization and blinding

If both knees were osteoarthritic, then at screening the investigator selected the knee that best satisfied the requirements for the study. In cases where both knees satisfied all inclusion and exclusion criteria, the study knee was selected based on greater baseline WOMAC A pain score. Patients were assigned to treatment by a sequential (by clinical site) randomization schedule in blocks of 4 following confirmation of eligibility before study medication was administered as a single intra-articular injection into the knee joint space (inferior lateral to the patella). Randomization was developed and maintained by an independent statistician. Treatments were provided in kits containing blinded study vials, syringes, needles and acetaminophen (rescue medication). The Sponsor, the investigator, and all study staff having a role in the day-to-day conduct of the study remained blinded to treatment.

### Interventions

A total of 329 patients with OA knee pain were randomized 1∶1∶1∶1 across 4 study arms: 4 mL LMWF-5A, 4 mL saline vehicle control, 10 mL LMWF-5A or 10 mL saline vehicle control.

The starting material of LMWF-5A, HSA purchased from OctaPharma (Lachen, Switzerland), was subjected to centrifugation/ultrafiltration under sterile conditions and the ultrafiltrate, containing species with a MW less than 5000 Da, was separated. The ultrafiltrate contained DA-DKP (approximately 50 – 200 µM) and the excipients (i.e. sodium caprylate and sodium acetyltryptophanate). The ultrafiltrate was transferred for aseptic filling, to afford sterile drug product. The control arm in this study was saline vehicle control, rather than a true ‘placebo’, as saline has been shown to induce significant pain relief, especially in trials involving intra-articular injections [17].

The clinical effects of treatment on OA were evaluated during clinic visits at 6 and 12 weeks and telephone contacts at 2, 4, 8 and 10 weeks, using the WOMAC® osteoarthritis Index 3.1 5-point Likert score, the Patient's Global Assessment of disease severity (PGA) using a 5-point Likert Score, and the amount of acetaminophen after intra-articular injection. Acetaminophen was supplied in 500 mg tablets at baseline as a rescue medication, and allowed as 1 tablet every 4 hours as needed.

Safety was evaluated by recording adverse events (through 24 hours post-dose and at all follow-up contacts), vital signs and physical examination results (baseline, weeks 6 and 12).

### Outcomes

The primary endpoint was the change in the WOMAC average pain subscore by 5-point Likert scale between baseline and week 12. Both the Likert and VAS scales are validated[18]; the Likert scale may be more favorable over the VAS version[16], [19].

Secondary endpoints included: the incidence and severity of AEs; change in the WOMAC average subscore by 5-point Likert scale for stiffness (WOMAC B subscore), physical function (WOMAC C subscore), and pain with movement and pain at rest (WOMAC A subscore questions 1–2 and 3–5, respectively); change in PGA; use of rescue analgesia (acetaminophen).

### Power and sample size

We estimated the sample size based on the mean difference in the WOMAC A pain change from baseline at week 12 using a 2-way ANOVA. The estimate was based on detecting a treatment difference of 1.0 and 0.5 for 10 ml and 4 ml volumes, respectively (with a common SD of 0.9) using a 2 tailed alpha of 0.05. We estimated a sample size of 80 patients into each study arm for a total of 320 patients in a 1∶1∶1∶1 ratio across all 4 study arms (4 mL vehicle control, 4 mL LMWF-5A, 10 mL vehicle control, 10 mL LMWF-5A) in order to achieve power of at least 80% to demonstrate both main effects (treatment effect, volume effect) or an interaction between the two main effects.

### Statistical analysis

The primary endpoint, change in WOMAC A Pain subscore between baseline and week 12, was analysed using analysis of covariance (ANCOVA) to test the main effects of LMWF-5A vs vehicle control and 4 mL vs 10 mL, and their interaction. The WOMAC A baseline measure was used as the covariate; clinical site effect was non-significant, and therefore was not included (p = 0.42). Residuals of the model were assessed for normalcy and heteroscedasticity to ensure that the model was appropriate.

The following secondary endpoints were evaluated between treatment groups using a mixed-effects repeated measures ANCOVA, adjusted for the respective baseline value: change in WOMAC B stiffness, WOMAC C physical function, WOMAC A pain at rest, WOMAC A pain with movement, and PGA. The mixed-effects repeated measures ANCOVA covariance structure was modelled as first-order autoregressive with subject as a repeated effect, and treatment arm and time included in the model; the interaction between treatment arm and time was removed if not significant. Amount of rescue medication (overall pill count use of acetaminophen) followed a non-normal distribution and was analysed using a Wilcoxon rank-sum test; the study-wide mean pill count was used for imputation of missing data in 29 patients.

Adverse events were examined in all patients who were randomized and received study medication; no safety data was imputed. All efficacy analyses were performed in the intent-to-treat (ITT) population, defined as all patients who were randomized, received study medication and had at least one post-baseline observation. The ITT population was analyzed as randomized; data were imputed using a last observation carried forward approach (4.9% of missing data were imputed). The primary endpoint was also examined in subgroups according to Kellgren Lawrence Grade, and prior IA knee injection for pain due to OA.

Statistical analyses were performed using SAS® software, version 9.1.3 or later (SAS Institute, Cary, NC). All analyses were defined *a priori* and performed in accordance with the study Protocol and the Statistical Analysis Plan. The protocol and Statistical Analysis Plan for this trial and supporting CONSORT checklist are available as supporting information; see Checklist S1, Protocol S1 and SAP S1.There was no adjustment for multiple comparison testing; the change from baseline to week 12 was considered the primary endpoint, and all other time points were supportive. Statistical significance was set at *p* value < 0.05 for all analyses.

## Results

### Subject disposition, baseline data

**Figure 1:**
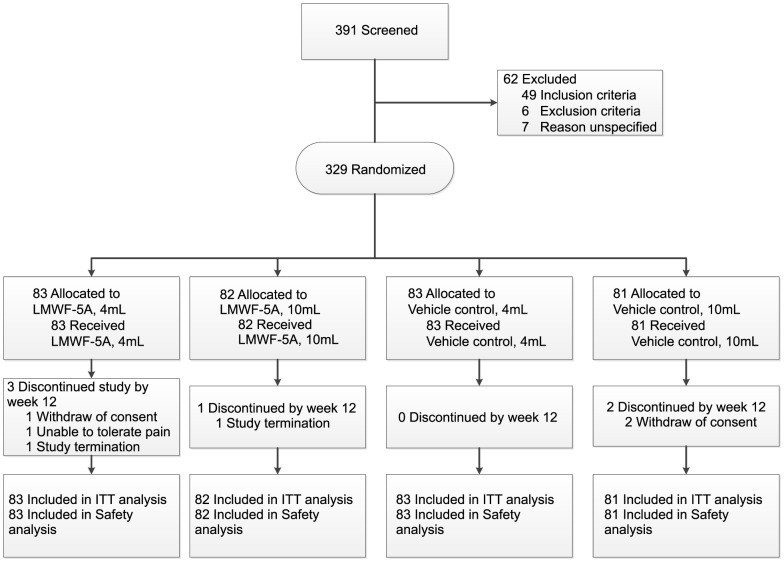
CONSORT Flow diagram.

Patient disposition can be seen in Figure 1, reported according to the CONSORT guidelines [20], [21]. A total of 329 patients were enrolled and received treatment, as follows: LMWF-5A 4 mL (n = 83), LMWF-5A 10 mL (n = 82) vehicle control 4 mL (n = 83) and vehicle control 10 mL (n = 81).

Baseline data is shown in Table 1. Enrolled patients ranged in age from 41 to 84 years, of whom 63.5% were female and 90.9% were white. The Kellgren Lawrence Grade was Grade II for 35%, III for 42%, and IV for 22%. There were no differences between treatment groups in demographic characteristics, baseline WOMAC scores or PGA.

**Table 1:** Demographics and baseline characteristics: ITT population.

	Randomized arms				Combined arms	
N (%)	Control, 4 mL (N = 83)	Control, 10 mL (N = 81)	LMWF-5A, 4 mL (N = 83)	LMWF-5A, 10 mL (N = 81)	Control (N = 164)	LMWF-5A (N = 165)
Female sex	57 (69%)	50 (62%)	56 (67%)	46 (56%)	107 (65%)	102 (62%)
White race	74 (89%)	77 (95%)	74 (89%)	74 (90%)	151 (92%)	148 (90%)
Hispanic	0 (0%)	2 (2%)	0 (0%)	2 (2%)	2 (1%)	2 (1%)
Age – Mean (SD)	60.7 (8.3)	63.8 (10.0)	62.7 (9.3)	62.8 (8.4)	62.2 (9.3)	62.7 (8.8)
BMI – Mean (SD)	34.5 (8.0)	32.1 (6.5)	33.2 (7.8)	32.8 (6.6)	33.3 (7.4)	33.0 (7.2)
Left study knee	42 (51%)	40 (49%)	35 (42%)	41 (50%)	82 (50%)	76 (46%)
Previous injection	58 (70%)	54 (67%)	49 (59%)	58 (71%)	112 (68%)	107 (65%)
Injection Type						
Steroid	32 (55%)	24 (44%)	25 (51%)	26 (45%)	56 (50%)	51 (48%)
Hyaluronic acid	20 (34%)	19 (35%)	17 (35%)	20 (34%)	39 (35%)	37 (35%)
Other	6 (10%)	11 (20%)	7 (14%)	12 (21%)	17 (15%)	19 (18%)
K-L Grade						
II	29 (35%)	26 (32%)	28 (34%)	32 (39%)	55 (34%)	60 (36%)
III	32 (39%)	34 (42%)	38 (46%)	35 (43%)	66 (40%)	73 (44%)
IV	22 (27%)	21 (26%)	17 (20%)	15 (18%)	42 (26%)	32 (19%)
PGA – Mean (SD)	3.4 (0.8)	3.4 (0.8)	3.4 (0.65)	3.4 (0.8)	3.4 (0.8)	3.4 (0.7)
WOMAC– Mean (SD)						
Pain	2.3 (0.5)	2.2 (0.6)	2.2 (0.5)	2.2 (0.5)	2.3 (0.5)	2.2 (0.5)
Stiffness	2.4 (0.8)	2.4 (0.8)	2.3 (0.7)	2.4 (0.8)	2.4 (0.8)	2.3 (0.8)
Function	2.3 (0.6)	2.2 (0.6)	2.1 (0.6)	2.2 (0.6)	2.2 (0.6)	2.2 (0.6)

Control, saline vehicle control; BMI, Body Mass Index; K-L Grade, Kellgren Lawrence Grade; PGA, Patient Global Assessment; WOMAC, Western Ontario and McMaster Universities Osteoarthritis Index.

The BMI is the weight in kilograms divided by the square of the height in meters. The PGA scores can range from 0 to 5. Scores for the WOMAC can range from 0 to 5.

### Treatment efficacy

**Figure 2:**
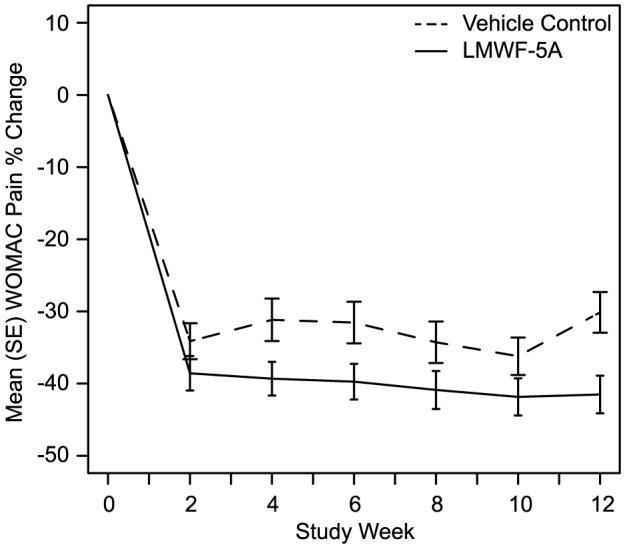
Summary of the percent improvement in the Western Ontario and McMaster Universities Osteoarthritis (WOMAC) pain subscore.

LMWF-5A resulted in a statistically significant improvement in pain as compared to vehicle control (−0.93 vs −0.72, respectively), Table 2. An injection volume effect was not observed (p = 0.64). The estimated difference from control was −0.25 (95% CI: −0.08 – −0.41), p = 0.004. The reduction in pain with LMWF-5A compared to vehicle control was observed as early as week 4 (p = 0.03), and persisted to week 12 (p = 0.004). The percent reduction in pain over time was significantly greater for LMWF-5A as compared to vehicle control (week 12: 42.3% and 31.7%, respectively), Figure 2.

**Table 2:** Summary of the Western Ontario and McMaster Universities Osteoarthritis Index (WOMAC) Mean Change in Pain (SE) over Time.

	Randomized arms				Combined arms		
Week	Control, 4 mL (n = 83)	Control, 10 mL (n = 81)	LMWF-5A, 4 mL (n = 83)	LMWF-5A, 10 mL (n = 82)	Control (n = 164)	LMWF-5A (n = 165)	p value†
Week 2	−0.75 (0.08)	−0.88 (0.10)	−0.86 (0.08)	−0.90 (0.08)	−0.81 (0.06)	−0.88 (0.06)	.14
Week 4	−0.68 (0.10)	−0.83 (0.10)	−0.84 (0.08)	−0.93 (0.07)	−0.76 (0.07)	−0.88 (0.05)	**.03**
Week 6*	−0.71 (0.09)	−0.82 (0.11)	−0.88 (0.08)	−0.91 (0.08)	−0.77 (0.07)	−0.89 (0.06)	**.04**
Week 8	−0.74 (0.09)	−0.89 (0.11)	−0.92 (0.09)	−0.94 (0.09)	−0.81 (0.07)	−0.93 (0.06)	.06
Week 10	−0.79 (0.08)	−0.91 (0.10)	−0.97 (0.08)	−0.90 (0.09)	−0.85 (0.06)	−0.94 (0.06)	.11
Week 12*	−0.71 (0.08)	−0.73 (0.11)	−0.93 (0.08)	−0.92 (0.09)	−0.72 (0.07)	−0.93 (0.06)	**.004**

Control: Saline vehicle control.

* Data were collected at in-person clinic visits; data at all other Weeks were collected via telephone.

† P values were calculated using ANCOVA, with adjustment for baseline WOMAC A pain score.

Patients treated with LMWF-5A demonstrated significant improvements in the following secondary endpoints, as compared to vehicle control: PGA (−0.87 vs −0.65, p = 0.01), physical function, (−0.78 vs −0.64, p = 0.04); pain at rest (−0.91 vs −0.70, p = 0.004); pain with movement (−0.96 vs −0.75, p = 0.01), Table 3. There were no differences in reduced stiffness between treatment groups. There was a trend towards a reduced number of acetaminophen pills used over the study period for LMWF-5A as compared to vehicle control (median (IQR)): 24.0 (0, 62) vs 34.0 (5, 85.5), p = 0.09.

**Table 3:** Summary of Additional Efficacy endpoints, reported as Mean Change (SE) at Week 12, ITT.

Mean (SE) change	Control, 4 mL (n = 83)	Control, 10 mL (n = 81)	LMWF-5A, 4 mL (n = 83)	LMWF-5A, 10 mL (n = 82)	Control (n = 164)	LMWF-5A (n = 165)	p value†
Secondary efficacy endpoint							
Stiffness*	−0.55 (0.10)	−0.80 (0.11)	−0.66 (0.10)	−0.79 (0.10)	−0.67 (0.08)	−0.72 (0.07)	.41
Physical function*	−0.58 (0.08)	−0.69 (0.11)	−0.72 (0.09)	−0.83 (0.09)	−0.64 (0.07)	−0.78 (0.06)	**.04**
Resting pain*	−0.71 (0.09)	−0.68 (0.11)	−0.90 (0.09)	−0.91 (0.09)	−0.70 (0.07)	−0.91 (0.06)	**.004**
Moving Pain*	−0.69 (0.09)	−0.81 (0.11)	−0.98 (0.09)	−0.94 (0.10)	−0.75 (0.07)	−0.96 (0.07)	**.01**
PGA subscale	−0.55 (0.12)	−0.74 (0.13)	−0.96 (0.11)	−0.77 (0.13)	−0.65 (0.09)	−0.87 (0.08)	**.01**
Subgroup population, WOMAC A Pain							
Prior injection	−0.66 (0.10)	−0.71 (0.12)	−0.81 (0.12)	−0.87 (0.11)	−0.68 (0.08)	−0.84 (0.08)	**.04**
No prior injection	−0.82 (0.15)	−0.77 (0.22)	−1.11 (0.11)	−1.05 (0.13)	−0.80 (0.13)	−1.09 (0.08)	.051
K-L Grade II	−0.83 (0.15)	−1.01 (0.22)	−0.94 (0.12)	−1.07 (0.14)	−0.92 (0.13)	−1.01 (0.09)	.70
K-L Grade III	−0.62 (0.11)	−0.76 (0.14)	−0.95 (0.13)	−0.82 (0.15)	−0.69 (0.09)	−0.89 (0.10)	**.04**
K-L Grade IV	−0.67 (0.19)	−0.34 (0.21)	−0.88 (0.22)	−0.83 (0.16)	−0.51 (0.14)	−0.86 (0.14)	**.02**

* WOMAC, Western Ontario and McMaster Universities Osteoarthritis.

Control: Saline vehicle control; Index. PGA, Patient Global Assessment of disease severity; K-L, Kellgren-Lawrence.

† P values were calculated using mixed-effects repeated measures ANCOVA, with adjustment for baseline score.

### Subgroup analyses

The effect of LMWF-5A was most pronounced in patients with severe knee OA (Table 3). In particular, LMWF-5A resulted in a significant improvement in pain in patients with severe OA (Kellgren Lawrence Grade IV), with an estimated difference from vehicle control of −0.42 (95% CI: −0.08 – −0.77), p = 0.02.

Patients with prior knee injection observed a significant improvement in mean pain with LMWF-5A as compared to vehicle control at week 12, while patients without prior knee injection saw a borderline significant improvement in pain with LMWF-5A as compared to vehicle control at week 12 (table 3).

### Safety

Adverse events were reported for 144 patients (44%), and were similar in patients who received LMWF-5A (41%) and vehicle control (47%), Table 4. Only arthralgia was reported in at least 5% of patients (7% LMWF-5A, 15% vehicle control). AEs were generally mild; severe AEs were observed in 5% and 6% of patients treated with LMWF-5A and vehicle control, respectively. There were no deaths and no AEs resulting in treatment change or study discontinuation. There were 7 reported SAEs (2% incidence in both treatment groups); no SAE was considered related to study drug.

**Table 4:** Summary of Adverse Events (AEs).

	Randomized arms				Combined arms	
n (%)	Control, 4 mL (n = 83)	Control, 10 mL (n = 81)	LMWF-5A, 4 mL (n = 83)	LMWF-5A, 10 mL (n = 82)	Control (n = 164)	LMWF-5A (n = 165)
Overall summary of AEs						
Any AE	41 (49%)	36 (44%)	35 (42%)	32 (39%)	77 (47%)	67 (41%)
Any Related AE	13 (16%)	8 (10%)	9 (11%)	8 (10%)	21 (13%)	17 (10%)
Any severe AE	5 (6%)	5 (6%)	5 (6%)	3 (4%)	10 (6%)	8 (5%)
Any SAE	2 (2%)	2 (2%)	0 (0%)	3 (4%)	4 (2%)	3 (2%)
Any Related SAE	0 (0%)	0 (0%)	0 (0%)	0 (0%)	0 (0%)	0 (0%)
Related AEs, by preferred term, descending order						
Arthralgia	7 (8%)	3 (4%)	3 (4%)	4 (5%)	10 (6%)	7 (4%)
Injection site pain	2 (2%)	2 (2%)	2 (2%)	1 (1%)	4 (2%)	3 (2%)
Headache	3 (4%)	1 (1%)	1 (1%)		4 (2%)	1 (< 1%)
Joint stiffness	2 (2%)		1 (1%)	2 (2%)	2 (1%)	3 (2%)
Joint swelling		1 (1%)		1 (1%)	2 (1%)	1 (< 1%)
Flushing	1 (1%)			1 (1%)	1 (< 1%)	1 (< 1%)
Nausea		1 (1%)	1 (1%)		1 (< 1%)	1 (< 1%)
Anxiety	1 (1%)				1 (< 1%)	
Injection site joint pain	1 (1%)				1 (< 1%)	
Muscle spasms	1 (1%)				1 (< 1%)	
Musculoskeletal discomfort	1 (1%)				1 (< 1%)	
Myalgia				1 (1%)		1 (< 1%)
Osteoarthritis			1 (1%)			1 (< 1%)
Urticaria				1 (1%)		1 (< 1%)

The percent of patients reporting treatment-related AEs was 10% with LMWF-5A and 13% with vehicle control; the most commonly occurring treatment-related AE was arthralgia (n = 17) and injection site pain (n = 7).

## Discussion

This randomized clinical trial demonstrated that a single intra-articular injection of LMWF-5A is safe and effective at both 4 mL and 10 mL volumes. Our study represents a potential major breakthrough in identifying a treatment for pain due to moderate to severe OA. Our trial's primary and secondary efficacy endpoints of improvements in pain, function, and overall assessment of disease severity support significant efficacy of LMWF-5A. The clinically and statistically significant reduction in study outcomes with LMWF-5A was observed after only a single IA injection into the knee. Both treatments were well tolerated, with a low incidence of treatment-related adverse events.

This is the first published study to evaluate an intra-articular injection of the low molecular weight fraction of HSA (LMWF-5A) to patients with OA. This low molecular weight fraction of HSA has been extensively evaluated *in vitro* and *in vivo* for its contents and its anti-inflammatory properties. One of the active ingredients, DA-DKP, has been shown to have multiple anti-inflammatory and immune modulating effects [12], [13], [15]. In a previous unpublished randomized controlled trial of 43 patients, LMWF-5A was efficacious at reducing pain in adults with OA of the knee, and was well tolerated with only minor treatment-related AEs reported. LMWF-5A resulted in a trend towards a significant decrease in overall pain NRS at 12 weeks compared to placebo (−1.6 vs. −0.36, p = 0.07), which became statistically significant when patients who received betamethasone rescue injection after week 1 were excluded (−2.22 vs. −0.46, p = 0.04).

Intra-articular corticosteroids have been used historically for the treatment of OA, but they are believed to be associated with significant safety [22] and efficacy limitations [4], [23]. Intra-articular hyaluronan products have been licensed in the USA for the treatment of knee OA since 1997. However, the magnitude of the therapeutic effects of IA hyaluronan products on OA of the knee is controversial due to low trial quality, publication bias and questionable efficacy [7], [24], [25], [26], [27], with inconclusive recommendations from the AHRQ [28] and a strong recommendation against their use by the AAOS [4].

Despite recommendations against their use, Hylan G-F 20 is the current treatment of choice in patients who cannot be managed with analgesics. In the pivotal trial of a single IA injection of 6 ml Hylan G-F 20, a borderline statistically significant reduction in pain was demonstrated, with an estimated difference from control of −0.15 (95% CI: −0.30 to −0.002), p = 0.047), corresponding to a 31.3% improvement in pain over 26 weeks in patients treated with Hylan G-F20. In comparison, our study demonstrated that a single IA injection of LMWF-5A resulted in a clinically and statistically significant reduction in pain, with an estimated difference from control at the study endpoint of −0.25 (95% CI: −0.41 to −0.08), p = 0.004, corresponding to a 42.3% improvement in pain at 12 weeks in patients treated with LMWF-5A. The accepted threshold for a minimum clinically important improvement (MCII), defined as the smallest change in a measurement that signifies important improvement in a patient's symptom, is −40.8% in WOMAC A pain change from baseline with knee OA, which only LMWF-5A exceeded [29].

Our results were even more robust in patients with severe OA (Kellgren-Lawrence Grade IV), with a non-significant treatment effect in patients with minimal OA (Kellgren-Lawrence Grade II), a finding worth discussing. The severity of disease (i.e. pathology at presentation) appeared to result in differing treatment effects in our study. We believe our results are partially due to a pronounced saline effect in patients with minimal OA (Grade II), resulting in a non-significant reduction in pain for patients treated with LMWF-5A over saline placebo in this subgroup. The effect of saline was less pronounced in patients with moderate-to-severe OA, resulting in a clinically and statistically significant reduction in pain with LMWF-5A over saline. We believe LMWF-5A represents an alternative IA treatment, providing relief for the pain of moderate to severe OA of the knee, particularly in patients with severe OA in whom there are currently no pharmacologic treatments.

The primary limitation is that our study was designed to follow patients to 12 weeks following IA injection, which may not capture the maximum difference between groups. Systematic reviews of intra-articular injections suggest that the primary endpoint of change in pain is generally measured at 13–26 weeks for IA hyaluronan products [25] and at 4 weeks for IA corticosteroids [23], in which the maximum treatment effect was demonstrated at 5–13 weeks for IA hyaluronan products [25], [30] and 2–3 weeks for IA corticosteroids [23]. In our study, patients treated with vehicle control observed an initial decrease in pain of −0.81 (36%) at week 2, which gradually worsened to −0.72 (32%) by week 12; on the contrary, patients treated with LMWF-5A observed an initial decrease in pain of −0.88 (40%) at week 2, with further decreases in pain of −0.93 (42%) by week 12. We are conducting an additional pivotal trial of LMWF-5A with a longer observation period of at least 20 weeks to identify maximum treatment effect. Secondarily, patients routinely present with bilateral OA, and it is likely that efficacy measures may be affected by pain due to OA of the contra lateral (non-treated) knee. We did not record the presence of bilateral OA in this study, and thus cannot determine whether the treatment effect differed between patients with unilateral vs. bilateral OA. Lastly, we included all patients with radiologic OA, defined by Kellgren Lawrence Grades II, III and IV [31]. Patients with varying stages of disease severity present with differing pathologies; the pathology at presentation may result in a different treatment effect at each severity grade. While this heterogeneity of our population may be considered a potential limitation, we believe this to also be a strength of our study, making it more generalizable than previously published studies of intra-articular injection for treatment of OA of the knee.

In conclusion, this randomized, controlled clinical trial demonstrated that a single intra-articular injection of LMWF-5A is a safe and effective treatment for osteoarthritis. Our results demonstrate improvements in pain and function, as well as overall disease severity with LMWF-5A as compared to vehicle control. These findings represent a potential major breakthrough in identifying a treatment for pain due to osteoarthritis, particularly in patients with severe osteoarthritis where no other safe and effective therapies exists prior to joint replacement.

## Supporting Information

Protocol S1 **Protocol AP-003A (Version 2.0, 15 May 2013).**
(PDF)

SAP S1 **Statistical Analysis Plan AP-003-A (Version 2.0, 8 August 2013).**
(PDF)

Checklist S1 **CONSORT 2010 checklist of information to include when reporting a randomised trial.**
(DOC)

Issued Patents S1 **Issued Patents for “AMPION”, as of December 5, 2013.**
(DOCX)
