# Supplementary material for: A Randomized Clinical Trial to Evaluate Two Doses of an Intra-Articular Injection of LMWF-5A in Adults with Pain Due to Osteoarthritis of the Knee
Source: PLoS One. 2014 Feb 3;9(2):e87910. doi: 10.1371/journal.pone.0087910 (PMC3912151; doi:10.1371/journal.pone.0087910)
Supplement: Protocol S1 — Protocol AP-003A (Version 2.0, 15 May 2013). (PDF) [file pone.0087910.s001.pdf]

CLINICAL STUDY PROTOCOL

A RANDOMIZED, PLACEBO-CONTROLLED, DOUBLE-BLIND STUDY TO EVALUATE THE EFFICACY AND SAFETY OF TWO DOSES OF INTRA-ARTICULAR INJECTION OF AMPION™ IN ADULTS WITH PAIN DUE TO OSTEOARTHRITIS OF THE KNEE

STUDY NUMBER: AP-003-A

**Drug Development Phase:**	Phase 2
**Investigational Product:**	Ampion™
**Indication:**	Osteoarthritis of the knee
**Sponsor:**	Ampio Pharmaceuticals, Inc.<br>5445 DTC Parkway Suite 925<br>Greenwood Village, CO 80111
**Lead Investigators:**	Matthew J. Phillips, MD<br><br>Brain McGrath, MD
**Date:**	Version 2.0 15 May 2013

Conduct: In accordance with the ethical principles that originate from the Declaration of Helsinki and that are consistent with International Conference on Harmonisation (ICH) guidelines on Good Clinical Practice (GCP) and regulatory requirements as applicable.

Confidential Information
This document is the sole property of Ampio Pharmaceuticals, Inc. This document and any and all information contained herein has to be considered and treated as strictly confidential. This document shall be used only for the purpose of the disclosure herein provided. No disclosure or publication shall be made without the prior written consent of Ampio Pharmaceuticals, Inc.

PROTOCOL SIGNATURE PAGE

I have read and understand the contents of this clinical protocol for Study No. AP-003-A dated 15 May 2013 and agree to meet all obligations of Ampio Pharmaceuticals, Inc. as detailed in all applicable regulations and guidelines.

Signed By:

<Study personnel signature> ______________________ **<Enter date>** ______________________

PROTOCOL SYNOPSIS

**Sponsor:**	**Investigational Product:**	**Developmental Phase:**
Ampio Pharmaceuticals, Inc.	Ampion™	Phase 2

Title of Study:

A Randomized, Placebo-Controlled, Double-Blind Study to Evaluate the Efficacy and Safety of a Two Doses of Intra-Articular Injection of Ampion™ in Adults with Pain Due to Osteoarthritis of the Knee

Protocol Number:

AP-003-A

Study Center(s):

Approximately 10 sites

Lead Investigators:

Matthew J. Phillips, MD and Brian McGrath, MD

Indication:

Pain of osteoarthritis (OA) of the knee

Number of patients:

320 total, 80 per study arm: 4mL Ampion™, 4mL placebo, 10mL Ampion™, 10mL placebo

Objectives:

The primary trial objective is to evaluate the greater efficacy of 10 mL Ampion™ versus 10 mL placebo than 4 mL Ampion™ versus 4 mL placebo intra-articular (IA) injection in improving knee pain, when applied to patients suffering from OA of the knee.

The secondary trial objectives include: evaluation of the safety of an intra-articular injection of Ampion™ when applied to patients suffering from OA of the knee, evaluation of the efficacy of intra-articular injection of Ampion™ and placebo on stiffness and function when applied to patients suffering from OA of the knee and evaluation of responder status defined by the Outcome Measures in Rheumatology Clinical Trials and Osteoarthritis Research Society International (OMERACT-OARSI) criteria.

Secondary exploratory objectives are to: analyze the effect, if any, of Ampion™ versus placebo on intra-articular inflammatory growth markers in a subset of patients and to assess any radiological changes visible in MRI.

**Sponsor:**	**Investigational Product:**	**Developmental Phase:**
Ampio Pharmaceuticals, Inc.	Ampion™	Phase 2

Methods:

Randomized, placebo-controlled, double-blind study with a 28 day screening period for each patient followed by a 12 week participation period. A total of 320 patients, 80 patients per study arm, with osteoarthritis knee pain will be randomized 1:1:1:1 across 4 study arms: 4mL placebo, 4mL Ampion™, 10mL placebo, and 10mL Ampion™. A subset of patients, approximately 20 total patients, randomized 1:1 from the 10mL injection arms will undergo clinical laboratory testing and a 1-2mL aspiration of synovial fluid of the index knee at Baseline and Week 12, and an MRI of the index knee at Baseline and Week 12.

The clinical effects of treatment on OA pain will be evaluated during clinic visits at 6 and 12 weeks, and telephone contacts at 2, 4, 8 and 10 weeks, using the Western Ontario and McMaster Universities Arthritis Index (WOMAC®) osteoarthritis Index 3.1, and the Patient's Global Assessment of disease severity (PGA). The WOMAC® is a validated pain scoring system and sets the standard for the patient response. In order not to bias the collection of data, only questions from the validated WOMAC pain scale will be asked of patients. Clinical meaningfulness will be determined by the end results of this trial, specifically by the apparent clinical benefit versus any adverse events or any increased apparent risk. Safety will be assessed by recording adverse events (through 24 hours post-dose and at all follow-up contacts) and physical examination and vitals (Baseline, Weeks 6 and 12).

Diagnosis and Main Criteria for Inclusion:

1. Able to provide written informed consent to participate in the study
2. Willing and able to comply with all study requirements and instructions of the site study staff
3. Male or female, 40 years to 85 years old (inclusive)
4. Must be ambulatory
5. Index knee must be symptomatic for greater than 6 months with a clinical diagnosis of OA and supported by radiological evidence (Kellgren Lawrence Grade II to IV) that is not older than 6 months prior to the date of screening
6. Moderate to moderately-severe OA pain in the index knee (rating of at least 1.5 on the WOMAC Index 3.1 5-point Likert Pain Subscale) assessed at screening
7. Moderate to moderately-severe OA pain in the index knee (even if chronic doses of nonsteroidal anti-inflammatory drug (NSAID), which have not changed in the 4 weeks prior to screening, have been/are being used)
8. No analgesia (including acetaminophen [paracetamol]) taken 12 hours prior to an efficacy measure
9. No known clinically significant liver abnormality (e.g. cirrhosis, transplant, etc.).

**Sponsor:**	**Investigational Product:**	**Developmental Phase:**
Ampio Pharmaceuticals, Inc.	Ampion™	Phase 2

Main Criteria for Exclusion:

1. As a result of medical review and screening investigation, the Principal Investigator considers the patient unfit for the study
2. A history of allergic reactions to human albumin (reaction to non-human albumin such as egg albumin is not an exclusion criterion)
3. A history of allergic reactions to excipients in 5% human albumin (N-acetyltryptophan, sodium caprylate)
4. Presence of tense effusions
5. Inflammatory or crystal arthropathies, acute fractures, history of aseptic necrosis or joint replacement in the affected knee, as assessed locally by the Principal Investigator
6. Isolated patella femoral syndrome, also known as chondromalacia
7. Any other disease or condition interfering with the free use and evaluation of the index knee for the duration of the trial (e.g. cancer, congenital defects, spine osteoarthritis)
8. Major injury to the index knee within the 12 months prior to screening
9. Severe hip osteoarthritis ipsilateral to the index knee
10. Any pain that could interfere with the assessment of index knee pain (e.g. pain in any other part of the lower extremities, pain radiating to the knee)
11. Any pharmacological or non-pharmacological treatment targeting OA started or changed during the 4 weeks prior to randomization or likely to be changed during the duration of the study
12. Use of the following medications:
    a. No IA injected pain medications in the study knee during the study
    b. No analgesics containing opioids. NSAIDs may be continued at levels preceding the study and acetaminophen is available as a rescue medication during the study from the provided supply
    c. No topical treatment on osteoarthritis index knee during the study
    d. No significant anticoagulant therapy (e.g. Heparin or Lovenox) during the study (treatment such as Aspirin and Plavix are allowed)
    e. No systemic treatments that may interfere with safety or efficacy assessments during the study
    f. No immunosuppressants
    g. No use of corticosteroids > 10 mg prednisolone equivalent per day (if ≤ 10 mg prednisolone, the dose must be stable)
13. No human albumin treatment in the 3 months before randomization or throughout the duration of the study

**Sponsor:** Ampio Pharmaceuticals, Inc.	**Investigational Product:** Ampion™	**Developmental Phase:** Phase 2
**Test Product, Dose and Mode of Administration:**<br><br>Ampion™, 4 mL or 10 mL, single intra-articular injection in the knee		
**Reference Therapy, Dose and Mode of Administration:**<br><br>Saline placebo, 4mL or 10 mL, single intra-articular injection in the knee		
**Study Duration:**<br><br>12 weeks		
**Criteria for Evaluation:**<br><br>Safety:<br>Adverse events, physical examination, and vitals<br><br>Efficacy:<br>WOMAC osteoarthritis Index 3.1 and PGA		
**Statistical Methods:**<br><br>The primary efficacy endpoint is the change from Baseline to Week 12 in the WOMAC Index 3.1 A pain subscore. The primary efficacy analysis will use the intent-to-treat (ITT) analysis population with the per-protocol (PP) analysis population as supportive, with comparisons between 10 mL Ampion™ injection vs 10 mL placebo injection and 4 mL Ampion™ injection vs 4 mL placebo injection, and 10mL Ampion™ injection vs 4 mL Ampion™ injection. Statistical analysis of WOMAC Index 3.1 pain scores will be by analysis of covariance with baseline WOMAC Index 3.1 as the only covariate.<br><br>There will be no statistical tests for incidence and severity of treatment emergent adverse events (TEAEs).		

TABLE OF CONTENTS

PROTOCOL SYNOPSIS 3

LIST OF ABBREVIATIONS AND DEFINITION OF TERMS 11

1 INTRODUCTION 13
1.1 Study Drug 13
1.2 Background to the Disease 13
1.3 Previous Human Experience 14
1.4 Preclinical Data 14
1.4.1 Pharmacology Studies 14
1.4.2 Toxicity and Safety Studies 14
1.5 Clinical Experience 14

2 RATIONALE FOR THE STUDY 18
2.1 Rationale for the Doses and the Dosing Regimen 18

3 STUDY DESIGN 19
3.1 Study Design Overview 19
3.2 STUDY OBJECTIVES 19
3.2.1 Primary Objective 19
3.2.2 Secondary Objectives 19
3.3 Safety Monitoring committee 20
3.4 Stopping Rules 20
3.5 Study Endpoints 20
3.5.1 Primary Endpoint 20
3.5.2 Secondary Endpoints 20
3.6 Blinding and Randomization 21
3.7 Code-breaking 21

4 SELECTION OF PATIENTS 22
4.1 Number of Patients 22
4.2 Recruitment Methods 22
4.3 Inclusion Criteria 22
4.4 Exclusion Criteria 23
4.5 Inclusion of Patients Incapable of Giving Informed Consent 24

5 STUDY PLAN AND PROCEDURES 25
5.1 Description of Study visits 25

5.1.1 Visit 1 (Day -28 through Day 1, in-clinic); Screening....25
5.1.2 Visit 2 (Day 1, in-clinic) Baseline/Randomization/Treatment....26
5.1.3 Visit 3 (Day 2, telephone contact)....26
5.1.4 Visit 4 (Week 2 ± 5 days, telephone contact)....26
5.1.5 Visit 5 (Week 4 ± 5 days, telephone contact)....26
5.1.6 Visit 6 (Week 6 ± 5 days, in-clinic)....27
5.1.7 Visit 7 (Week 8 ± 5 days, telephone contact)....27
5.1.8 Visit 8 (Week 10 ± 5 days, telephone contact)....27
5.1.9 Visit 9 (Week 12 ± 5 days, in-clinic)....27
5.1.10 Early Termination Visit....28
5.1.11 Unscheduled Visits....28
5.1.12 Missed Visits....28
5.1.13 CONCOMITANT MEDICATIONS....29

6 METHODS OF ASSESSMENT....30
6.1 Efficacy Assessments....30
6.1.1 WOMAC® Osteoarthritis Index (Bellamy 1988)....30
6.1.2 Patient's Global Assessment of Disease Severity (PGA)....31
6.2 Safety Parameters....31
6.2.1 Vital Signs....31
6.2.2 Aspiration of the index knee....31
6.2.3 Clinical laboratory tests....32
6.2.4 Magnetic Resonance Imaging....33

7 DISCONTINUATION CRITERIA....35
7.1 Early Discontinuation of the Study....35
7.2 Early Discontinuation of Individual Patients....35

8 TREATMENT....36
8.1 Dosing and administration of study medication....36
8.2 Drug Storage and Accountability....36
8.3 Concomitant Treatments and rescue medication....37
8.4 Treatment Compliance....38

9 ADVERSE EVENTS....39
9.1 Definition of an adverse event....39
9.2 Definition of a Serious Adverse Event....39
9.3 Recording of adverse events and serious adverse events....40

9.3.1 AE Follow-up....40
9.3.2 Overdose....41
9.4 Serious Adverse Event Reporting....41
9.4.1 Reporting Requirements....41
9.4.2 SAE Contact Information....41

10 STATISTICAL METHODS....42
10.1 GENERAL CONSIDERATIONS....42
10.1.1 Statistical and Analytical Plan:....42
10.2 STUDY OBJECTIVES....42
10.3 ANALYSIS POPULATIONS....42
10.3.1 Safety Analysis Population:....42
10.3.2 Intent-to-treat Population:....43
10.3.3 Per Protocol Population:....43
10.3.4 Statistical Analysis of Primary Effectiveness Endpoint....43
10.3.5 Primary Hypotheses....43
10.3.6 Definition of Study Visits....44
10.3.7 Number of patients to receive study drug....44
10.3.8 Disposition of patients....44
10.3.9 Interim analysis....44
10.3.10 Blinding and randomization....44
10.3.11 Data presentation....45
10.3.11.1 Demographic and Baseline Characteristics:....45
10.3.11.2 Medical History and Physical Examination:....45
10.3.11.3 Concomitant Medications or Treatments:....45
10.3.11.4 Safety data:....45
10.3.11.5 Efficacy data:....46
10.4 STUDY ENDPOINTS....46
10.4.1 Primary Endpoint....46
10.4.2 Secondary Endpoints....47
10.5 Missing and Spurious Data....48

11 REGULATORY, ETHICAL AND LEGAL OBLIGATIONS....49
11.1 Declaration of Helsinki....49
11.2 Good Clinical Practice....49
11.3 Institutional Review Boards/Ethics Committees....49
11.4 Regulatory Authority Approval....49
11.5 Informed Consent....49

11.6 Patient Confidentiality and Disclosure 49
11.7 Collection, Monitoring and Auditing Study Documentation, and Data Storage 50
11.7.1 Collection of Data and Monitoring Procedures 50
11.7.2 Auditing Procedure 51
11.7.3 Retention of Documents 51
11.8 Disclosure of Information 51
11.9 Discontinuation of the Study 51
11.10 Study Report, Publication Policy and Archiving of Study Documentation 51
11.10.1 Study Report and Publication Policy 51
11.10.2 Study Documents 52
11.10.3 Archiving of Documents 52

12 REFERENCES 53

13 APPENDICES 55
13.1 Contact List 56

LIST OF IN-TEXT TABLES

Table 6.1 Biomarker testing 33
Table 6.2 Schedule of Assessments and Procedures 34
Table 9.1 Definitions of AE Severity 39
Table 9.2 Guidelines for Determining the Relationship (if any) Between Adverse Event and the Study Drug 39

LIST OF ABBREVIATIONS AND DEFINITION OF TERMS

Abbreviation	Definition
°C	degrees Celsius
°F	degrees Fahrenheit
μg	microgram
AE	adverse event
ALP	alkaline phosphatase
ALT	alanine transaminase (SGPT)
ARDS	adult respiratory distress syndrome
AST	aspartate transaminase (SGOT)
BP	Blood pressure
CFR	Code of Federal Regulations
CI	confidence interval
cm	centimeter
CRF	case report form
CRO	Clinical Research Organization
Da	dalton
DA-DKP	Asp-Ala diketopiperazine
dL	deciliter
eDC	electronic data capture
FDA	Food and Drug Administration
g	11.26 x $(RPM/1000)^2$ x Radius (cm)
GCP	Good Clinical Practice
GGT	gamma-glutamyl transferase
GI	gastrointestinal
HSA	human serum albumin
IA	intra-articular
ICF	informed consent form
ICH	International Conference on Harmonisation
IEC	Independent Ethics Committee
IRB	Institutional Review Board
ITT	intent-to-treat
kDa	kilodalton
LDH	lactic dehydrogenase
MCH	mean cell hemoglobin
MCHC	mean cell hemoglobin concentration
MCV	mean cell volume
mg	milligram
mL	milliliter
NA	not applicable
ng	nanogram
NSAID	non-steroidal anti-inflammatory drug
OA	osteoarthritis
OMERACT-OARSI	outcome measures in rheumatology clinical trials and osteoarthritis research society international
PGA	patient's global assessment of disease severity

Abbreviation	Definition
PP	per protocol population
Radius	Distance (in Centimeters) from the center of rotation to the bottom of tube in the rotor
RBC	red blood cell
REB	Research Ethics Board
RPM	Rounds Per Minute
SAE	serious adverse event
SD	standard deviation
SEM	standard error of the mean
SMC	Safety Monitoring Committee
SOP	standard operation procedure
TEAE	treatment-emergent adverse event
TEER	Trans Endothelial Electrical Resistance
WBC	white blood cell
WCC	white cell count
WO	washout
WOMAC	Western Ontario and McMaster Universities Arthritis Index
WORMS	Whole-Organ MRI Score

1 INTRODUCTION

Ampion™, the < 5 kilodalton (kDa) ultrafiltrate of 5% human serum albumin (HSA), is being developed to provide short term relief for the pain of moderate to severe osteoarthritis of the knee, in patients who have failed to respond adequately to medications such as acetaminophen or other nonsteroidal anti-inflammatory drugs (NSAIDs). Bar-Or et al found that commercial preparations of HSA are heterogeneous in terms of posttranslational modifications, including C- and N-terminal truncation, cysteinylation, glycation and nitrosylation (Bar-Or D, 2005). An important modification is the cleavage of the two N-terminal amino acids Aspartate (D) and Alanine (A). This occurs by the action of naturally occurring dipeptidases existing free in serum and bound to or associated with leukocytes (Ohnuma 2006), and during heat-treatment of commercial preparations of HSA. After cleavage, the dipeptide undergoes cyclisation to form a diketopiperazine (aspartylalanyl diketopiperazine [DA-DKP]). DA-DKP is found in the low molecular weight fraction (< 3000 Da) of commercial lots of HSA at concentrations ranging from approximately 50–200 μM. Studies *in vitro* and in patients suggest that it is an active ingredient in the pharmacological effects of HSA and of Ampion™.

HSA has a long history of clinical use as a colloid replacement therapy, dating back over 60 years. Currently, HSA is approved for the indications hypovolemia, hypoalbuminemia, prevention of central volume depletion after paracentesis due to cirrhotic ascites, ovarian hyperstimulation, adult respiratory distress syndrome (ARDS), acute nephrosis, and hemolytic disease of the newborn. In addition to its effects on oncotic pressure, HSA has pharmacological effects including decreased inflammation (Quinlan 2005), decreased vascular permeability (Evans 2002) and reduction in the production of pro-inflammatory cytokines (Bar-Or 2006, Shimonkevitz 2008) and no adverse effect on cardiac safety (Vincent JL, 2004).

1.1 STUDY DRUG

Ampion™ is the < 5 kDa ultrafiltrate of 5% HSA.

1.2 BACKGROUND TO THE DISEASE

Osteoarthritis (OA) is the most common form of arthritis, affecting 25–35 million people in the US. Symptomatic OA of the knee occurs in 10–13% of individuals over the age of 60. OA is caused by inflammation of the soft tissue and bony structures of the joint which worsens over time and leads to progressive thinning of articular cartilage, narrowing of the joint space, synovial membrane thickening, osteophyte formation and increased density of subchondral bone. These changes eventually result in chronic pain and disability, and deterioration of the joint despite drug therapy may require eventual surgery for total joint replacement.

Current drug treatment for OA of the knee relies on pain control with analgesics, and anti-inflammatory treatment with NSAIDs and intra-articular injections of steroids or hyaluronates. The current drug treatments have been shown to have mixed results and may have significant limitations due to various adverse effects such as gastrointestinal irritation

and bleeding. Therefore there is a need for additional anti-inflammatory drug treatments for OA, particularly as the population ages and the prevalence of obesity, a contributing factor to the development of OA, continues to rise (Guccione 1994, Felson 1988, Dillon 2006, Zhang 2010).

1.3 PREVIOUS HUMAN EXPERIENCE

In addition to the extensive clinical experience with HSA, Ampio Pharmaceuticals, Inc. has completed a Phase Ib and Phase II randomized, controlled, repeat-blind study of a single intra-articular (IA) injection of Ampion™ < 5 kDa ultrafiltrate of 5% HSA in adults with OA of the knee. The study is summarized in Section 1.5.

1.4 PRECLINICAL DATA

1.4.1 Pharmacology Studies

In vitro pharmacology studies demonstrated that a low molecular weight fraction (< 3000 daltons [Da]) of more than five commercial preparations of HSA, and a cyclized dipeptide contained in that fraction, Asp-Ala diketopiperazine (DA-DKP) have a range of anti-inflammatory properties, which may be expected to ameliorate the symptoms of inflammation, including pain, in man (Bar-Or et al 2006). These studies showed reduced inflammatory cytokine production from human T lymphocytes *in vitro* via modulation of signal transduction through increased expression of Rap-1, and inhibition of macrophage activation. In addition, DA-DKP enhanced integrity of human endothelial cell junctions in the transendothelial electrical resistance (TEER) assay, which would result in decreased vascular permeability to the influx of inflammatory cells *in vivo*. No pharmacokinetics studies were performed as the plasma levels of the constituents of the < 5000 Da preparation, including DA-DKP, after intra-articular injection of a therapeutic dose in man or in laboratory animals are anticipated to be below the limits of detection. For more information, consult the Investigator Brochure.

1.4.2 Toxicity and Safety Studies

HSA and other components of serum albumin preparations are species specific and may be expected to be immunogenic if repeatedly injected into non-human species, even at very low concentrations. For this reason, it was not possible to perform formal toxicological studies in animals.

1.5 CLINICAL EXPERIENCE

Ampio Pharmaceuticals, Inc. has completed a Phase Ib and Phase II randomized, controlled, double-blind study of a single intra-articular injection of Ampion™ < 5 kDa ultrafiltrate of 5% HSA in adults with OA of the knee (AIK study). This study was completed at a single study center in Adelaide, Australia in 2012.

The study was conducted in 2 parts. In Part A (phase Ib), a total of 60 patients each received a single injection totaling 10mL in one knee of one of the following treatments:

- Treatment A: Ampion™ combined with betamethasone/lignocaine suspension
- Treatment B: Ampion™ combined with betamethasone/saline suspension
- Treatment C: Saline placebo combined with betamethasone/lignocaine suspension.

Two sentinel patients (1 active and 1 placebo) were dosed 24 hours before the remaining patients were randomized and dosed.

In Part B (phase II), a total of 42 patients each received a single 4 mL injection in one knee of one of the following treatments:

- Treatment D: Ampion™
- Treatment E: Saline placebo.

Safety was measured by recording the incidence of adverse events (AEs), vital signs at pre-dose and study Day 4, 12-lead ECG readings at Screening and 24 hours post-dose and clinical blood safety tests (biochemistry, hematology) at Screening, and 24 hours post-dose.

Efficacy was assessed as follows:

Part A

Primary Endpoint

- The change from pre-injection baseline of the pain numerical rating scale at 6, 24 and 72 hours post-dose.

Secondary Endpoints

- The change from pre-injection baseline in WOMAC® Osteoarthritis Index 3.1 (complete scale, pain subscore, stiffness subscore and function subscore) at 24 and 72 hours after intra-articular injection.
- The change from pre-injection baseline for requirement for rescue medications (acetaminophen [paracetamol]) to 24 hours and 72 hours after intra-articular injection.
- The change from pre-injection baseline of the range of motion (degrees of flexion and extension in the study knee) for patients with limited range of motion due to pain and inflammation, as assessed by the Investigator or nominee at 24 and 72 hours post-dose.
- Incidence of treatment-emergent adverse events (TEAEs).

Part B

Primary Endpoint

- The change from pre-injection baseline of the pain numerical rating scale at 6, 24 and 72 hours post-dose, and at Day 8, Day 30 and Day 84 post-dose.

Secondary Endpoints

- The change from pre-injection baseline in WOMAC® Osteoarthritis Index 3.1 (complete scale, pain subscore, stiffness subscore and function subscore) at 24 and 72 hours after intra-articular injection.
- The change from pre-injection baseline for requirement for rescue medications (acetaminophen [paracetamol]) to 24 hours and 72 hours after intra-articular injection.
- Changes over time in mobility at Day 8, Day 30 and Day 84 post-dose compared with pre-dose and the immediate post-dose period.
- Incidence of TEAEs, including clinically significant changes in laboratory test results, vital signs, and ECG findings.

Safety was assessed by recording adverse events, vital signs, twelve lead ECG readings, and clinical blood safety tests (biochemistry, hematology), and concomitant medications.

Results

Efficacy:

Overall pain (as assessed by the pain numerical rating score) and WOMAC scores were reduced post-dose for each of the treatment groups for the duration of the study ($p < 0.05$), except placebo at Day 84.

There were no statistically significant differences between changes from baseline in pain NRS or WOMAC scores for patients who received Ampion™ (with betamethasone/lignocaine or with betamethasone/saline) compared with patients who did not receive Ampion™ (saline with betamethasone/lignocaine) and between patients who received Ampion™ compared with patients who received saline. However, for Part B only there was a trend in a significant difference between changes from baseline at Day 30 and at Day 84 for patients who received Ampion™ compared to patients who received saline placebo (Day 30: $p = 0.12$; Day 84: $p = 0.07$). This trend became statistically significant at Day 84 in patients who did not receive rescue medication ($p = 0.04$).

For Part B only, there was a trend towards a higher percentage of responders at Day 84 for patients receiving Ampion™ vs. Placebo ($p = 0.06$).

For patients who received Ampion™ in Parts A and B of the study, there was no apparent difference between patients who received Ampion™ with betamethasone (with or without lignocaine) or Ampion™ alone.

Use of acetaminophen rescue medication up to 72 hours post-dose was highest in the Treatment E group (saline).

Safety:

In Part A of this study, TEAEs were reported for 17 of the 60 patients (28%) following dose administration, with a total of 31 TEAEs. In Part B of this study, TEAEs were reported for 20 of the 43 patients (47%) following dose administration, with a total of 27 AEs. In Part B, a higher proportion of TEAEs was reported in patients who received saline (12 patients, 57%) compared with patients who received Ampion™ (8 patients, 36%).

In Part A, AEs deemed to be related to study drug administration (definitely, possibly and probably) were reported in 4 of 60 patients (7%) and included headache, joint swelling and joint stiffness of the knee and flushing of the face. In Part B, TEAEs deemed to be related to study drug administration (possibly) were reported in 3 of 43 patients (7%) and included headache and joint swelling of the knee.

Commonly occurring AEs were headache, joint swelling and stiffness in the knee. AEs of moderate severity included headache, oropharyngeal pain, back pain, joint injury, vessel puncture site hematoma and hypertension, all of which were assessed as not related to study drug.

There were no deaths or other serious TEAEs.

There was a trend for white cell count (WCC), neutrophils, and monocytes to be increased at 24 hours post-dose in patients who received Ampion™ (with betamethasone/lignocaine and with betamethasone/saline) and saline with betamethasone/lignocaine in Part A of the study. WCC, neutrophils and monocytes were unchanged in Part B of the study. There was also a trend for lymphocytes to be decreased at 24 hours post-dose in Part A of the study, with no change in lymphocytes in Part B.

There were no clinically significant differences in safety as assessed by biochemistry clinical laboratory tests, vital signs, and ECG assessments between treatments.

In conclusion, pain (as assessed by the pain NRS) and WOMAC scores were reduced post-dose for each of the treatment groups for the duration of the study, except placebo at Day 84, with no significant differences between treatment groups. However, trends for long-term reductions in pain were observed for patients who received Ampion™ compared with patients who received saline.

Ampion™ was considered safe and well tolerated at the dose used in the study.

2 RATIONALE FOR THE STUDY

This randomized blinded study is a Phase 2 trial designed to follow on from the completed Phase Ib and II studies and further evaluate the efficacy and safety of two doses of IA injection of Ampion™ in adult patients with pain due to OA of the knee.

2.1 RATIONALE FOR THE DOSES AND THE DOSING REGIMEN

This trial will compare the 4mL volume of Ampion™ used in the Phase Ib and II studies to a 10mL volume, which is routinely used for IA injections to the knee.

3 STUDY DESIGN

3.1 STUDY DESIGN OVERVIEW

This is a randomized, placebo-controlled, double-blind study with a 28 day screening period for each patient followed by a 12 week participation period. A total of 320 patients, 80 patients per study arm, with OA knee pain will be randomized 1:1:1:1 across 4 study arms: 4mL Ampion™, 4mL placebo, 10mL Ampion™ or 10mL placebo. A subset of patients, approximately 20 total patients, randomized 1:1 from the 10mL injection arms will undergo a 1-2mL aspiration of the index knee (Baseline and Week 12) and MRI (Baseline and Week 12).

The clinical effects of treatment on OA pain will be evaluated during clinic visits at 6 and 12 weeks and telephone contacts at 2, 4, 8 and 10 weeks, using the Western Ontario and McMaster Universities Arthritis Index (WOMAC®) osteoarthritis Index 3.1, and the Patient's Global Assessment of disease severity (PGA). The WOMAC® is a validated pain scoring system and sets the standard for the patient response. In order not to bias the collection of data, only questions from the validated WOMAC pain scale will be asked of patients. Clinical meaningfulness will be determined by the end results of this trial, specifically by the apparent clinical benefit versus any adverse events or any increased apparent risk. Safety will be assessed by recording adverse events (through 24 hours post-dose and at all follow-up contacts) and physical examination and vitals (Baseline, Weeks 6 and 12), and clinical laboratory tests for the 10mL study arms (Baseline, Weeks 6 and 12).

3.2 STUDY OBJECTIVES

3.2.1 Primary Objective

The primary trial objective is to evaluate whether the efficacy of 10 mL Ampion™ versus 10 mL placebo is greater than the efficacy of 4 mL Ampion™ versus 4 mL placebo intra-articular (IA) injection in improving knee pain, when administered to patients suffering from OA of the knee.

3.2.2 Secondary Objectives

The secondary trial objectives include: evaluation of the safety of an intra-articular injection of Ampion™ when applied to patients suffering from OA of the knee, evaluation of the efficacy of intra-articular injection of Ampion™ and placebo on stiffness and function when applied to patients suffering from OA of the knee and evaluation of responder status defined by the Outcome Measures in Rheumatology Clinical Trials and Osteoarthritis Research Society International (OMERACT-OARSI) criteria.

Secondary exploratory objectives are to: analyze the effect, if any, of Ampion™ versus saline on intra-articular inflammatory growth markers in a subset of patients and to assess any radiological changes visible in MRI.

3.3 SAFETY MONITORING COMMITTEE

An internal Safety Monitoring Committee (SMC) will be established to review the safety of Ampion™ as the study progresses. The SMC will consist of independent clinicians not involved in the clinical trial, the Medical Monitor and Ampio Pharmaceuticals, Inc. representatives. The SMC will be primarily responsible for reviewing any serious Adverse Event (SAE) and other clinically important safety findings (e.g., discontinuations due to AEs) that may occur during the study. Prior to unblinding any treatment assignments, a charter will be developed to detail the SMC review responsibilities, the frequency of meetings, and data evaluation plans.

3.4 STOPPING RULES

The entire study may be stopped under defined circumstances as outlined in Section 7.

3.5 STUDY ENDPOINTS

3.5.1 Primary Endpoint

- Change in the WOMAC A pain subscore by 5-point Likert scale between baseline and Week 12

3.5.2 Secondary Endpoints

- Change in WOMAC A pain subscore between baseline and Weeks 2, 4, 6, 8, and 10
- Change in WOMAC B stiffness subscore between baseline and Weeks 2, 4, 6, 8, 10 and 12
- Change in WOMAC C physical function subscore between baseline and Weeks 2, 4, 6, 8, 10 and 12
- Change in PGA between baseline and Weeks 6, 8, 10 and 12
- Response status based on the OMERACT-OARSI criteria at Weeks 2, 4, 6, 8, 10 and 12
- Change in WOMAC A pain subscore questions 1 and 2 (pain with movement) between baseline and Weeks 2, 4, 6, 8, 10 and 12
- Change in WOMAC A pain subscore questions 3−5 (resting pain) between baseline and Weeks 2, 4, 6, 8, 10 and 12
- Use of rescue analgesia (amount of acetaminophen used)
- Incidence and severity of treatment-emergent adverse events (TEAEs)

3.6 BLINDING AND RANDOMIZATION

Patients will be assigned to treatment by a randomization schedule developed and maintained by an independent statistician. Ampion™ and the placebo will be provided in blinded study vials labeled with the appropriate information and packed into patient kits. Each patient kit will contain labeled vials, blinded for drug content, syringes, needles and rescue medication. Patient kits will be labeled with double-panel labels, blinded for drug content.

The Sponsor, the investigator, and all study staff having a role in the day-to-day conduct of the Study will remain blinded to treatment.

A comprehensive presentation of the data management and statistical analysis plan will be approved by Ampio Pharmaceuticals, Inc. prior to unblinding of study data.

3.7 CODE-BREAKING

Where required, safety personnel and/or investigator may be unblinded to a particular patient's treatment assignment to meet reporting requirements to Regulators. Unblinding may occur by unmasking the double-panel label that contains the treatment disclosure panel.

4 SELECTION OF PATIENTS

4.1 NUMBER OF PATIENTS

A total of approximately 320 patients will be enrolled in the study in a 1:1:1:1 ratio across all 4 study arms (single injection 4mL placebo, single injection 4mL Ampion™, single injection 10mL placebo, single injection 10mL Ampion™). 80 total patients will be randomized into each study arm to achieve the statistically significant amount of 75 patients per treatment arm. Blood samples will be collected for all patients (Baseline, Weeks 6 and 12) randomized into the 10mL injection study arms. A subset of patients, approximately 20 total patients (10 patients from the 10mL placebo study arm and 10 patients from the 10mL Ampion™ study arm), will be assessed for exploratory secondary endpoints and will receive an MRI (Baseline and Week 12) and knee aspiration (Baseline and Week 12).

For the analysis of, saline v Ampion™ (by 4mL v 10mL) treatment effects assumed are a SD of 0.9 for the WOMAC A pain score with mean pain decreases of 2.0 for 10mL Ampion™, 1.5 for 4mL Ampion™, and 1.0 for both saline placebo arms. With enrollment of 75 for each of 4 arms, the power to demonstrate both main effects or in interaction between the two main effects is greater than 80% using 2-tailed alpha of 0.05.

4.2 RECRUITMENT METHODS

Patients will be recruited from the population being seen by Investigators at the clinical sites participating in the study. In addition, notifications about the opportunity for patients to participate in a clinical trial will be sent to referring physicians. A description of the clinical trial will also be posted at ClinicalTrials.gov, and advertisements and/or other notices may be produced to advise potential study patients on how they may obtain information about study participation. All such materials will be reviewed and approved by the Institutional Review Board (IRB) prior to their publication or dissemination.

4.3 INCLUSION CRITERIA

It is recommended that patients should have a WOMAC 5-point Likert Pain Subscale <1.5 in the contralateral knee, which is assessed at screening. Patients should fulfill all of the following inclusion criteria:

1. Able to provide written informed consent to participate in the study
2. Willing and able to comply with all study requirements and instructions of the site study staff
3. Male or female, 40 years to 85 years old (inclusive)
4. Must be ambulatory
5. Index knee must be symptomatic for greater than 6 months with a clinical diagnosis of OA and supported by radiological evidence (Kellgren Lawrence Grade II to IV) that is not older than 6 months prior to the date of screening
6. Moderate to moderately-severe OA pain in the index knee (rating of at least 1.5 on the WOMAC Index 3.1 5-point Likert Pain Subscale) assessed at screening

7. Moderate to moderately-severe OA pain in the index knee (even if chronic doses of nonsteroidal anti-inflammatory drug (NSAID), which have not changed in the 4 weeks prior to screening, have been/are being used)
8. No analgesia (including acetaminophen [paracetamol]) taken 12 hours prior to an efficacy measure
9. No known clinically significant liver abnormality (e.g. cirrhosis, transplant, etc.)

4.4 EXCLUSION CRITERIA

Patients fulfilling one or more of the following criteria may not be enrolled in the study:

1. As a result of medical review and screening investigation, the Principal Investigator considers the patient unfit for the study
2. A history of allergic reactions to human albumin (reaction to non-human albumin such as egg albumin is not an exclusion criterion)
3. A history of allergic reactions to excipients in 5% human albumin (N-acetyltryptophan, sodium caprylate)
4. Presence of tense effusions
5. Inflammatory or crystal arthropathies, acute fractures, history of aseptic necrosis or joint replacement in the affected knee, as assessed locally by the Principal Investigator
6. Isolated patella femoral syndrome, also known as chondromalacia
7. Any other disease or condition interfering with the free use and evaluation of the index knee for the duration of the trial (e.g. cancer, congenital defects, spine OA)
8. Major injury to the index knee within the 12 months prior to screening
9. Severe hip OA ipsilateral to the index knee
10. Any pain that could interfere with the assessment of index knee pain (e.g. pain in any other part of the lower extremities, pain radiating to the knee)
11. Any pharmacological or non-pharmacological treatment targeting OA started or changed during the 4 weeks prior to randomization or likely to be changed during the duration of the study
12. Use of the following medications:
    a. No IA injected pain medications in the study knee during the study
    b. No analgesics containing opioids. NSAIDs may be continued at levels preceding the study and acetaminophen is available as a rescue medication during the study from the provided supply
    c. No topical treatment on osteoarthritis index knee during the study
    d. No significant anticoagulant therapy (e.g. Heparin or Lovenox) during the study (treatment such as Aspirin and Plavix are allowed)
    e. No systemic treatments that may interfere with safety or efficacy assessments during the study
    f. No immunosuppressants
    g. No use of corticosteroids > 10 mg prednisolone equivalent per day (if ≤ 10 mg prednisolone, the dose must be stable)
13. Any human albumin treatment in the 3 months before randomization.

4.5 INCLUSION OF PATIENTS INCAPABLE OF GIVING INFORMED CONSENT

No patient incapable of giving informed consent may be enrolled in the study.

5 STUDY PLAN AND PROCEDURES

Patients will be randomized into a study arm and receive an IA injection of either Ampion™ or saline placebo on Visit 2, Day 1. Telephone contact will be made with the patient 24 hours after the IA injection. The patient will be followed for 12 weeks and the clinical effects of treatment on OA pain will be evaluated during clinic visits at 6 and 12 weeks, and during telephone contacts at 2, 4, 8 and 10 weeks, using the WOMAC osteoarthritis Index 3.1 (pain subscore, stiffness subscore and function subscore) and an overall global severity assessment (Patient's Global Assessment [PGA]). Safety will be assessed by recording adverse events (through 24 hours post-dose and at all follow-up in-clinic visits and telephone contacts), vital signs (Baseline, and Weeks 6 and 12), recording prior and concomitant medications including start/stop dates, indication, dose and frequency (through 24 hours post-dose and at all follow-up in-clinic visits and telephone contacts) and physical examination (Baseline and Weeks 6 and 12). The assessments and procedures performed at each patient visit or contact are described in Section 5.1 and in Table 6.2 Schedule of Assessments and Procedures.

5.1 DESCRIPTION OF STUDY VISITS

5.1.1 Visit 1 (Day -28 through Day 1, in-clinic); Screening

The following procedures will be performed at Visit 1 (Screening):

- Obtain written informed consent before the start of any study specific procedure.
- Review medical history including all previous treatments for OA.
- Record prior and concomitant medications including start/stop dates, indication, dose and frequency.
- Record demographic data including year of birth, age, gender and race.
- Measure and record height and weight.
- Perform and record physical examination.
- Record Kellgren Lawrence Grade from radiological assessment that is not older than 6 months prior to screening. Patient may have an x-ray at screening in order to satisfy this requirement.
- Record pain in the index knee with the WOMAC Index 3.1.
- Record pain in the contralateral knee with the WOMAC Index 3.1 pain subsection
- Record vital signs. (Measure body temperature, systolic / diastolic blood pressure [BP] and pulse rate).
- Evaluate all inclusion and exclusion criteria to ensure that patients meet all inclusion criteria and none of the exclusion criteria.

5.1.2 Visit 2 (Day 1, in-clinic) Baseline/Randomization/Treatment

The following procedures will be performed at Visit 2 (Baseline/Randomization/Treatment):

- Confirm eligibility (review inclusion/exclusion criteria).
- Randomize patient to study arm.
- Perform and record physical examination.
- Record vital signs. (Measure body temperature, systolic / diastolic blood pressure [BP] and pulse rate) pre- and post-injection.
- Perform WOMAC and PGA evaluations prior to treatment.
- For patients randomized into a 10mL injection study arm: Collect blood samples pre-intra-articular injection.
- For patients randomized into the subset of 20 patients in the 10mL injection study arm: Obtain MRI of the index knee pre-intra-articular injection.
- For patients randomized into the subset of 20 patients in the 10mL injection study arm: Aspirate the index knee pre-intra-articular injection.
- Perform intra-articular injection of study drug.
- Record post-injection AEs if observed.
- Record concomitant medications.
- Issue rescue medication (acetaminophen [paracetamol]).

5.1.3 Visit 3 (Day 2, telephone contact)

The following procedures will be performed at Visit 3:

- Record AEs.
- Record concomitant medications.
- Review rescue medication use.

5.1.4 Visit 4 (Week 2 ± 5 days, telephone contact)

The following procedures will be performed at Visit 4:

- Perform WOMAC and PGA evaluations of the study knee.
- Record AEs.
- Record concomitant medications.
- Review rescue medication use.

5.1.5 Visit 5 (Week 4 ± 5 days, telephone contact)

The following procedures will be performed at Visit 5:

- Perform WOMAC and PGA evaluations of the study knee.
- Record AEs.
- Record concomitant medications.
- Review rescue medication use.

5.1.6 Visit 6 (Week 6 ± 5 days, in-clinic)

The following procedures will be performed at Visit 6:

- Perform and record physical examination.
- Record vital signs. (Measure body temperature, systolic / diastolic blood pressure [BP] and pulse rate).
- Perform WOMAC and PGA evaluations of the study knee.
- Record AEs.
- Record concomitant medications.
- Review rescue medication use.
- For patients randomized into a 10mL injection study arm: Collect blood samples.

5.1.7 Visit 7 (Week 8 ± 5 days, telephone contact)

The following procedures will be performed at Visit 7:

- Perform WOMAC and PGA evaluations of the study knee.
- Record AEs.
- Record concomitant medications.
- Review rescue medication use.

5.1.8 Visit 8 (Week 10 ± 5 days, telephone contact)

The following procedures will be performed at Visit 8:

- Perform WOMAC and PGA evaluations of the study knee.
- Record AEs.
- Record concomitant medications.
- Review rescue medication use.

5.1.9 Visit 9 (Week 12 ± 5 days, in-clinic)

The following procedures will be performed at Visit 9:

- Perform and record physical examination.
- Record vital signs. (Measure body temperature, systolic / diastolic blood pressure [BP] and pulse rate).
- Perform WOMAC and PGA evaluations of the study knee.
- Record AEs.
- Record concomitant medications.
- Review rescue medication use.
- For patients randomized into a 10mL injection study arm: Collect blood samples.
- For patients randomized into the subset of 20 patients in the 10mL injection study arm: Obtain MRI of the index knee.

- For patients randomized into the subset of 20 patients in the 10mL injection study arm: Aspirate the index knee. *Note: if a patient has a 'dry tap', where no fluid is obtained from the joint, at Baseline, then a Week 12 aspiration will not be performed.

5.1.10 Early Termination Visit

The following procedures will be performed at Early Termination Visit:

- Perform and record physical examination.
- Record vital signs. (Measure body temperature, systolic / diastolic blood pressure [BP] and pulse rate).
- Perform WOMAC and PGA evaluations of the study knee.
- Record AEs.
- Record concomitant medications.
- Review rescue medication use.
- For patients randomized into a 10mL injection study arm: Collect blood samples.

5.1.11 Unscheduled Visits

Additional visits may be scheduled at the discretion of the Investigator, for example as part of follow-up of AEs. Unscheduled Visits will follow the visit structure described in Section 5.1.9 for the Week 6 visit.

5.1.12 Missed Visits

Patients unable to complete a study visit as scheduled should be re-scheduled for a replacement visit as soon as possible. If a subject misses any scheduled follow-up visit and cannot be seen prior to the start of the visit range for the next follow-up visit, the visit is considered missed.

5.1.13 CONCOMITANT MEDICATIONS

The following medications / therapies are NOT allowed during this clinical study:

1. No IA injected pain medications in the study knee during the study.
2. No analgesics containing opioids. NSAIDs may be continued at levels preceding the study and acetaminophen is available as a rescue medication during the study from the provided supply.
3. No non-pharmacological treatment targeting OA started or changed during the study.
4. No topical treatment on osteoarthritis index knee during the study.
5. No significant anticoagulant therapy (e.g. Heparin or Lovenox) during the study (treatment such as Aspirin or Plavix is allowed)
6. No systemic treatments that may interfere with safety or efficacy assessments during the study.
7. No immunosuppressants.
8. No use of corticosteroids > 10 mg prednisolone equivalent per day (if ≤ 10 mg prednisolone, the dose must be stable).
9. No human albumin treatment during the study.

6 METHODS OF ASSESSMENT

Demographic Data

At Visit 1 (Screening), patient demographic data will be collected. These include: year of birth, age, gender and race.

Medical History

At Visit 1 (Screening) a complete medical history, including prior interventions to the study knee, will be obtained from each patient.

Concomitant Medications

Detailed history of medications will be documented for each patient at Visit 1 (Screening) and Visit 2 (Baseline). Concomitant medications (especially any changes in medication) will be documented for each patient at each scheduled visit.

Physical Examination and Vital signs

Height in feet and inches will be measured at Visit 1 (Screening).

Body weight in pounds (lb) will be measured at Visit 1 (Screening).

Body temperature (deg F) will be measured at each visit.

Systolic and diastolic BP and pulse rate will be measured with the patient in a seated position.

The full physical examination will consist of examining the following body systems: cardiovascular, respiratory, abdominal, skin and musculoskeletal other than the knee. The physical examination of the target knee will consist of evaluating the knee joint for effusion and tenderness on palpation. Table 6.2 Schedule of Assessments and Procedures

below lists the various study activities and timing.

6.1 EFFICACY ASSESSMENTS

Note: Efficacy questionnaire questions will be asked "with reference to study knee" i.e. to obtain scores specific for the treated knee.

6.1.1 WOMAC® Osteoarthritis Index (Bellamy 1988)

WOMAC Index 3.1 should be completed by patients at Screening, pre-dose and pre-aspiration as applicable on Day 1 (pre-dose and pre-aspiration, as applicable) and at Weeks 2, 4, 6 (prior to aspiration as applicable), 8, 10 and 12 (prior to aspiration as applicable). All patients are required to take at least 5 minutes to complete the questionnaire.

Patients are asked about their pain, stiffness, and function in the knee (study joint) due to arthritis during the last 48 hours.

Patients respond to each subscale by using a 5-point adjectival Likert score (0 = none, 1 = mild, 2 = moderate, 3 = severe, 4 = extreme).

6.1.2 Patient's Global Assessment of Disease Severity (PGA)

The PGA should be completed by patients at Screening, pre-dose Day 1 (prior to injection), and at Weeks 2, 4, 6, 8, 10 and 12.

Patients are asked the following question: "Considering all the ways in which your arthritis affects you, please indicate how you are doing."

Patients respond by using a 5-point adjectival Likert score (0 = very well, 1 = well, 2 = fair, 3 = poor, 4 = very poor).

6.2 SAFETY PARAMETERS

Based on results of a Phase Ib and a Phase II randomized, controlled, repeat-blind study of a single intra-articular injection of Ampion™ < 5 kDa ultrafiltrate of 5% HSA in adults with OA of the knee (AIK study), in which no clinically significant differences between active and placebo were found in either electrocardiogram or blood parameters, safety will be assessed by recording adverse events, vital signs, results of physical examination, and by recording prior and concomitant medications.

A subset of patients will undergo knee aspiration, clinical laboratory tests and MRI to assess the secondary exploratory objective of assessing the effect of Ampion™ versus saline on intra-articular inflammatory growth markers and to assess any radiological changes visible in MRI.

6.2.1 Vital Signs

Vital signs (radial pulse rate, blood pressure, and body temperature) should be recorded at Screening, pre- and post–treatment Day 1, and then on visits at Weeks 6 and 12.

Vital signs should be taken after the patient has rested in a seated position for at least 5 minutes.

6.2.2 Aspiration of the index knee

Aspiration of the index knee for biomarker analysis will be performed on a subset of approximately 20 total patients randomized 1:1 into 10mL placebo and Ampion™ injection study arms. Aspiration of the index knee will be performed at Baseline and Week 12 after WOMAC Index 3.1 and PGA assessment is performed before any, if any, treatment injection is performed (Baseline). The date of aspiration should be recorded in the study source notes/eCRF.

Aspiration should be performed according to the Principal Investigator's standard of care. It is recommended that aspiration is performed under sterile prep conditions, (i.e. prior to aspiration, the knee should be cleaned with an antiseptic) to a patient who is in a supine position with the index knee at extension. The area of aspiration is just posterior to the superior lateral corner of the patella. The treatment area may be anesthetized with a topical anesthesia, injection of lidocaine or nothing, as determined by the Principal Investigator. The recommended needle of choice for this injection is a 20 gauge needle that is 1.5 inches long. The needle may be passed obliquely towards the trochlear groove. Approximately 1-2 mL of synovial fluid will be aspirated and the volume of synovial fluid may be measured via the syringe. It is recommended that the synovial fluid is transferred into labeled tubes and placed in an upright position and frozen immediately at below -70°C, or below -20°C if the samples are frozen for less than 3 months. Synovial fluid will be assessed for cartilage formation and degradation using biomarker analysis.

Injection of study drug will occur after aspiration. Injection of study drug should proceed easily. Failure to easily inject should be documented as a potential non-inter-articular injection. The date of administration should be recorded in the study source notes/eCRF.

6.2.3 Clinical laboratory tests

Patients in the 10mL injection study treatment arms will undergo a blood draw at Baseline, Weeks 6 and 12 (or termination visit) for serological and hematology assessment. Blood draws may occur up to 24 hours before the scheduled visit date. Blood draws will be performed in compliance with standard laboratory procedures. Laboratory parameters for which clinically significant values are noted will be followed up at the Investigator's discretion. The standard precaution for venipuncture should be observed. Hemolysis should be avoided. It is recommended that 1 blood sample of at least 3.5mL be collected using a plain tube with separator gel and then gently inverted 5 times to mix clot activator with the blood. It is recommended that blood clot for 30 minutes at room temperature in a vertical position. Hemolysis should be avoided. If freezing is necessary, the labeled tubes will be placed in an upright position and frozen immediately and the interval between blood drawing and freezing should be less than approximately 1 ½ hours (90 minutes).

The following analysis will be conducted:

Serum biochemistry: Sodium, potassium, chloride, bicarbonate, glucose, urea, creatinine, creatine kinase, urate, phosphate, total calcium, cholesterol, albumin, globulins, protein, total bilirubin, conjugated bilirubin, gamma glutamyltransferase (GGT), alkaline phosphatase (ALP), alanine aminotransferase (ALT), aspartate aminotransferase (AST) and lactate dehydrogenase (LD).

Hematology: Hemoglobin, red blood cell count, hematocrit, mean cell volume (MCV), mean cell hemoglobin (MCH), mean cell hemoglobin concentration (MCHC), platelets, white cell count, neutrophils, lymphocytes, monocytes, eosinophils, and basophils.

Analysis with exploratory biomarkers, such as those listed in Table 6.1, may also be analyzed using either serum or synovial fluid.

TABLE 6.1 BIOMARKER TESTING

CARTILAGE FORMATION			
**Marker ID**	**Marker Name**	**Matrix**	**Analyze**
CS846	Chondroitin sulfate 846 Epitope	Serum and/or Synovial Fluid	Manual ELISA
PIIANP	Procollagen type IIA N-propeptide	Serum	Manual ELISA
**CARTILAGE DEGRADATION**			
**Marker ID**	**Marker Name**	**Matrix**	**Analyze**
COMP	Cartilage Oligomeric matrix Protein	Serum and/or Synovial Fluid	Manual ELISA
C2M	Matrix Metalloproteinase generated fragment of type II collagen	Serum	Manual ELISA

6.2.4 Magnetic Resonance Imaging

A magnetic resonance image (MRI) will be acquired for the subset of approximately 20 patients at Baseline and Week 12. MRI may occur up to 24 hours before the scheduled visit date. The Whole-Organ MRI Score (WORMS) system, or similar assessment, will be used to assess osteoarthritis of the knee and articular cartilage volume quantification using MRI (Peterfy et al, 2004).

TABLE 6.2 SCHEDULE OF ASSESSMENTS AND PROCEDURES

	Screening	Baseline Randomization Treatment	Post-treatment check (telephone contact)	Week 2 (telephone contact)	Week 4 (telephone contact)	Week 6	Week 8 (telephone contact)	Week 10 (telephone contact)	Week 12 Final Visit	Early Termination
**Visit #<br>Day #**	**1<br>Day-28 to 1**	**2<br>Day 1**	**3<br>Day 2**	**4<br>Day 14 ± 5**	**5<br>Day 28 ± 5**	**6<br>Day 42 ± 5**	**7<br>Day 56 ± 5**	**8<br>Day 70 ±5**	**9<br>Day 84 ± 5**	
Informed Consent	X									
Inclusion/exclusion criteria	X									
Medical history/prior medications	X									
Concomitant medications	X	X	X	X	X	X	X	X	X	X
Physical examination	X	X				X			X	X
Vital Signs	X	X				X			X	X
Randomization		X								
WOMAC	X	X		X	X	X	X	X	X	X
Patient's global assessment (PGA)	X	X		X	X	X	X	X	X	X
X-ray[1]	X									
MRI (WORMS)[2]		X							X	
Knee aspiration[3]		X							X	
Clinical laboratory tests[4]		X				X			X	X
Treatment with study drug		X								
Rescue medication		X								
Review Rescue medication			X	X	X	X	X	X	X	X
Adverse Events		X	X	X	X	X	X	X	X	X

Visits are in clinic except for Days 2 and Weeks 2, 4, 8 and 10 when patients will be contacted by telephone

[1] X-ray may be acquired at Screening to satisfy inclusion criteria, "Index knee must be symptomatic for greater than 6 months with a clinical diagnosis of OA and supported by radiological evidence (Kellgren Lawrence Grade II to IV) that is not older than 6 months prior to the date of screening".

[2] Only applicable for patients randomized into the subset of 20 patients in the 10mL injection study arms.

[3] Only applicable for patients randomized into the subset of 20 patients in the 10mL injection study arms.

[4] Only applicable for patients randomized into the 10mL injection study arms.

7 DISCONTINUATION CRITERIA

7.1 EARLY DISCONTINUATION OF THE STUDY

It is agreed that for reasonable cause, either the investigator or the Sponsor may terminate this study, provided a written notice is submitted at a reasonable time in advance of intended termination; if by the investigator notice is to be submitted to Ampio Pharmaceuticals, Inc., and if by the Sponsor, notice will be provided to each investigator.

If a severe local reaction or drug-related SAE occurs at any time during the study, the Safety Monitoring Committee will review the case immediately.

The study will be immediately suspended and no additional Ampion™ treatments administered pending review and discussion of all appropriate study data by the SMC if 1 or more patients develop any of the following adverse events deemed to be possibly, probably, or definitely related to Ampion™ by the Investigator and/or Medical Monitor, based upon close temporal relationship or other factors:

- Death
- Anaphylaxis (angioedema, hypotension, shock, bronchospasm, hypoxia, or respiratory distress)Induction of autoimmune arthritis
- Hepatic failure
- Aplastic anemia.

The study will not be restarted until all parties have agreed to the course of action to be taken and the IRB/EC has been notified.

7.2 EARLY DISCONTINUATION OF INDIVIDUAL PATIENTS

Patients are to be withdrawn from the study for any of the following reasons:

- Withdrawal of informed consent
- Patient is lost to follow-up.

Patients will also be withdrawn at any time if the investigator concludes that it would be in the patient's best interest for any reason. Protocol violations do not lead to patient withdrawal unless they constitute a significant risk to the patient's safety.

Patients can voluntarily withdraw from the trial for any reason at any time. They are to be considered withdrawn if they state an intention to withdraw, fail to return for visits, or become lost to follow up for any reason. Patients withdrawing from the study because of an AE should be followed for at least 30 days, resolution of the AE or until no further improvement is expected, whichever comes first.

8 TREATMENT

Eligible patients will receive a single intra-articular injection into one knee of either Ampion™ or saline placebo at a volume of either 4ml or 10ml, dependent on study arm.

Patients will be allocated to a sequentially numbered treatment in accordance with the randomization schedule following confirmation of eligibility before treatment.

8.1 DOSING AND ADMINISTRATION OF STUDY MEDICATION

Appropriately trained site personnel should administer the study treatment.

If both knees are osteoarthritic, then at Screening the Investigator should select the knee that best satisfies the requirements for the study (see inclusion criteria 6 - 7) as the study knee. At the time of dose administration, the study knee should be treated with received investigational product in accordance with the randomization schedule. The other knee should receive standard of care.

It is recommended that the study treatment be administered as an injection into the knee joint space under sterile prep conditions, (i.e. prior to injection, the knee should be cleaned with an antiseptic) to a patient who is in the seated position with the treatment knee flexed at 0 . The area of injection is inferior lateral to the patella; lateral level of the joint line. The Principal Investigator may determine whether anesthesia of the treatment area with a topical anesthesia, injection of lidocaine or nothing is appropriate. The recommended needle of choice for this injection is a 20 gauge needle that is 1.5 inches long. The needle may be passed through the fat pad to the firm surface of the intercondylar notch. Following the withdrawal of the needle, it is recommended that fingertip pressure be applied to the injection site, and then a sterile dressing (BandAid) is used to cover the injection site. Injection should proceed easily. Failure to easily inject should be documented as a potential non-inter-articular injection. The date of administration should be recorded in the study source notes/eCRF.

8.2 DRUG STORAGE AND ACCOUNTABILITY

Study drug should be stored at room temperature (59º – 77ºF or 15º – 25ºC) in a secure area with restricted access. Temperature monitors, and instructions to download the temperature data to Ampio Pharmaceuticals, Inc., will be included with each site shipment of study drug.

The Investigator, the clinical site pharmacist, or other personnel authorized to store and dispense investigational product is responsible for ensuring that the investigational product used in the clinical study is securely maintained as specified by the Sponsor and in accordance with the applicable regulatory requirements.

All investigational product is to be dispensed in accordance with the Investigator’s prescription and it is the Investigator’s responsibility to ensure that an accurate record is maintained of investigational product issued and returned.

If any quality issue is noticed upon the receipt or use of an investigational product (i.e. deficiencies in condition, packaging, appearance, associated documentation, labeling, expiry date, etc.), Ampio Pharmaceuticals, Inc. must be promptly notified.

Under no circumstances may the Investigator supply investigational product to a third party, allow the investigational product to be used other than as directed by this clinical study protocol, or dispose of investigational product in any other manner.

All investigational product not used during the study must be returned to Ampio Pharmaceuticals Inc., or designated representative after study completion. All empty vials of study drug must be returned to Ampio Pharmaceuticals, Inc., or its designated representative at the completion of the clinical trial.

8.3 CONCOMITANT TREATMENTS AND RESCUE MEDICATION

<u>The following medications / therapies are NOT allowed during this clinical study:</u>

1. No IA injected pain medications in the study knee during the study.
2. No analgesics containing opioids. NSAIDs may be continued at levels preceding the study and acetaminophen is available as a rescue medication during the study from the provided supply.
3. No non-pharmacological treatment targeting OA started or changed during the study.
4. No topical treatment on osteoarthritis index knee during the study.
5. No significant anticoagulant therapy (e.g. Heparin or Lovenox) during the study (treatment such as Aspirin or Plavix is allowed)
6. No systemic treatments that may interfere with safety or efficacy assessments during the study.
7. No immunosuppressants.
8. No use of corticosteroids > 10 mg prednisolone equivalent per day (if ≤ 10 mg prednisolone, the dose must be stable).
9. No human albumin treatment during the study.

Any medication used during the study should be recorded. All concomitant medication start and stop dates, total daily dose, route and indication should to be recorded.

The only allowed rescue medication is 500 mg of acetaminophen (paracetamol), 1 tablet every 4 hours as required by the patient.

8.4 TREATMENT COMPLIANCE

The injection of study drug will be performed by the Investigator or a designated member of the site clinical staff. A 5mL syringe will be included in patient kits for those patients randomized into the 4mL injection study arms. A 10mL syringe will be included in patient kits for the patients randomized into the 10mL injection study arms. 20 gauge needle(s) will be included in every patient kit. Compliance with treatment is thus assured.

9 ADVERSE EVENTS

9.1 DEFINITION OF AN ADVERSE EVENT

An adverse event (AE) is defined as any undesired medical occurrence in a patient or clinical investigation patient receiving a pharmaceutical product and which does not necessarily have a causal relationship with this treatment. An AE can therefore be any unfavorable sign and unintended sign (including an abnormal laboratory finding), symptom, or disease temporarily associated with the use of a study drug, whether or not related to the study drug.

Assessment of severity of an AE will be rated according to categories listed in TABLE 9.1.

TABLE 9.1 DEFINITIONS OF AE SEVERITY

**Grade 1 (MILD):**<br>The symptom is barely noticeable to the study patient and does not influence performance or functioning. Concomitant medication is not ordinarily indicated for relief of mild AEs.
**Grade 2 (MODERATE):**<br>The symptom is of sufficient severity to make the study patient uncomfortable and to influence performance of daily activities. Concomitant medication may be indicated for relief of moderate AEs.
**Grade 3 (SEVERE):**<br>The symptom causes severe discomfort, sometimes of such severity that the study patient cannot continue in the study. Daily activities are significantly impaired or prevented by the symptom. Concomitant medication may be indicated for relief of severe AEs.

Determination of the relationship between the AE and the study drug will be made using the guidelines presented in TABLE 9.2.

TABLE 9.2 GUIDELINES FOR DETERMINING THE RELATIONSHIP (IF ANY) BETWEEN ADVERSE EVENT AND THE STUDY DRUG

**Unrelated**	The adverse event is unlikely to have been caused by study drug.
**Possibly related**	It is unclear whether the adverse event may have been caused by study drug.
**Related**	The adverse event is likely to have been caused by study drug.

9.2 DEFINITION OF A SERIOUS ADVERSE EVENT

A Serious Adverse Event (SAE) is any untoward medical occurrence that occurs at any dose that:

- Results in death
- Is life-threatening (patient is at immediate risk of death from the event as it occurred)
- Requires in-patient hospitalization (formal admission to a hospital for medical reasons) or prolongation of existing hospitalization
- Results in persistent or significant disability/incapacity
- Results in a congenital anomaly/birth defect

Important medical events that may not result in death, are not life-threatening, or do not require hospitalization may be considered SAEs when, based on appropriate medical judgment, they may jeopardize the patient and may require medical or surgical intervention to prevent one of the outcomes listed in this definition. Examples of such events include allergic bronchospasm requiring intensive treatment in an emergency room or at home, blood dyscrasias or convulsions that do not result in in-patient hospitalization, or the development of drug dependency or drug abuse.

Hospitalizations for elective surgery or other medical procedures that are not related to a treatment-emergent AE are not considered SAEs.

9.3 RECORDING OF ADVERSE EVENTS AND SERIOUS ADVERSE EVENTS

Recording and reporting of adverse events should be in accordance with the FDA final "Guidance for Industry and Investigators Safety Reporting Requirements for INDs and BA/BE Studies" of December 2012.

Any AE is to be recorded in the eCRF. In order to avoid vague, ambiguous, or colloquial expressions, the AE should be recorded in standard medical terminology rather than the patient's own words. Whenever possible, the investigator should combine signs and symptoms that constitute a single diagnosis.

The existence of an AE may be concluded from a spontaneous report of the patient; from the physical examination; or from special tests e.g., laboratory assessments, where applicable, or other study-specified tests (source of AE).

The reporting period begins from the time that the patient receives IA injection at the Baseline visit through including patient's Final Visit at 12 weeks. Any events continuing at study exit will be followed for 30 days or to resolution, or until no improvement is expected, whichever comes first. Any SAE occurring after the reporting period must be promptly reported if a causal relationship to the investigational drug is suspected. If the patient begins a new therapy, the safety reporting period ends at the time the new treatment is started, however, death must always be reported when it occurs during the 12 week study period irrespective of intervening treatment.

Each AE is to be evaluated for duration, severity, seriousness, and causal relationship to the investigational drug. The action taken and the outcome must also be recorded.

9.3.1 AE Follow-up

All AEs occurring during the study are to be followed up in accordance with good medical practice until they are resolved, stabilized or judged no longer clinically significant or, if a chronic condition, until fully characterized. Any AEs that are considered drug-related (possibly related, definitely related) must be followed for 30 days, or to resolution, or until no improvement is expected, whichever occurs first.

9.3.2 Overdose

No information on treatment of overdose of Ampion™ is currently available. In the case of overdose the patient should be followed as for an AE and appropriate supportive medical treatment instigated.

9.4 SERIOUS ADVERSE EVENT REPORTING

9.4.1 Reporting Requirements

Unexpected serious suspected adverse reactions are subject to expedited reporting to FDA. ALL SAEs must be entered into the eCRF within 24 hours of first knowledge of the event by study personnel. It is important that the investigator provide his/her assessment of relationship to study drug at the time of the initial report. Entry of an SAE into the eCRF triggers an automatic alert to the CRO safety team. The following information must be reported:

- Protocol number
- Site and/or Investigator number
- Patient number
- Demographic data
- Brief description of the event
- Onset date and time
- Resolution date and time, if the event resolved
- Current status, if event not yet resolved
- Any concomitant treatment and medication
- Investigator's assessment of whether the SAE was related to Investigative product or not.

The CRO Safety Associate will contact the site for clarification of data entered onto the eCRF, or to obtain missing information. In the event of questions regarding SAE reporting, the site may contact the appropriate individual as in Section 9.4.2.

9.4.2 SAE Contact Information

Vaughan Clift, MD

Dedicated lines for SAE reporting:

Tel: 720-746-8605
Fax: 720-437-6501

Ampio Pharmaceuticals Inc, or their designee CRO, is responsible for submitting reports of AEs associated with the use of the drug that are both serious and unexpected to FDA according to 21 CFR 312.32 and the final guidance (2012). All investigators participating in ongoing clinical studies with the study medication will receive copies of these reports for prompt submission to their Institutional Review Board (IRB) or Ethics committee (EC).

10 STATISTICAL METHODS

10.1 GENERAL CONSIDERATIONS

This section describes the rules and conventions to be used in the presentation and analysis of the data. A comprehensive presentation of the data management and statistical analysis plan will be approved by Ampio Pharmaceuticals, Inc. prior to unblinding of study data.

10.1.1 Statistical and Analytical Plan:

This is a randomized, placebo-controlled, double-blind study to evaluate the efficacy and safety of treatment with intra-articular Ampion™ in patients with OA of the knee. Patients will be followed over a 12 week treatment period to assess the efficacy of Ampion™ on changes in pain, stiffness and function, and to assess the safety of Ampion™ by monitoring adverse events.

Efficacy variables will be assessed at Baseline and Weeks 2, 4, 6, 8, 10 and 12. Safety will be assessed by recording AEs at all visits, recording concomitant medication, physical assessment and vitals.

10.2 STUDY OBJECTIVES

The primary trial objective is to evaluate the greater efficacy of 10 mL Ampion™ versus 10 mL placebo than 4 mL Ampion™ versus 4 mL placebo intra-articular (IA) injection in improving knee pain, when applied to patients suffering from OA of the knee.

The secondary trial objectives include: evaluation of the safety of an intra-articular injection of Ampion™ when applied to patients suffering from OA of the knee, evaluation of the efficacy of intra-articular injection of Ampion™ and placebo on stiffness and function when applied to patients suffering from OA of the knee and evaluation of responder status defined by the Outcome Measures in Rheumatology Clinical Trials and Osteoarthritis Research Society International (OMERACT-OARSI) criteria.

Secondary exploratory objectives are to: analyze the effect, if any, of Ampion™ versus saline on intra-articular inflammatory growth markers in a subset of patients and to assess any radiological changes visible in MRI.

10.3 ANALYSIS POPULATIONS

10.3.1 Safety Analysis Population:

The safety analysis population is defined as all patients who are randomized and receive study medication (Ampion™ or placebo). Patients will be analyzed as treated.

10.3.2 Intent-to-treat Population:

The intent-to-treat (ITT) analysis population is defined as all patients who are randomized, receive study medication (Ampion™ or placebo) and have at least one post-baseline observation. All efficacy analyses will be performed in the ITT population. Patients will be analyzed as randomized.

10.3.3 Per Protocol Population:

The per protocol analysis population is defined as all patients included in the ITT analysis who met all entry criteria and had no major protocol violations. All efficacy analyses will be repeated in the per-protocol population. These analyses will be supportive of the ITT analysis. Patients will be analyzed as treated.

10.3.4 Statistical Analysis of Primary Effectiveness Endpoint

A 2x2 analysis of covariance will be used to test the main effects of Ampion™ v saline and 4mL v 10mL, and their interaction. The covariate will be the WOMAC A baseline measure. The main effect for Ampion™ v saline will address the question of the superiority of the active formulation. The superiority of the 10mL Ampion™ dose over the 4 mL Ampion™ dose may be demonstrated by either the presence of both significant main effects in which Ampion™ is better than saline and 10 mL is better than 4 mL or by showing by significant interaction that the two main effects act non-additively to enhance the combined effect of 10mL with Ampion™.

10.3.5 Primary Hypotheses

- Reduction in Pain, as measured by the WOMAC A pain subscore by 5-point Likert scale between baseline and Week 12 will be greater in patients treated with Ampion™ than with saline placebo:

Null Hypothesis: $\mu A = \mu P$
Alternate Hypothesis: $\mu A < \mu P$

- Reduction in Pain, as measured by the WOMAC A pain subscore by 5-point Likert scale between baseline and Week 12 will be greater in patients treated with Ampion™ 10mL than with Ampion™4mL:

Null Hypothesis: $\mu A10 = \mu P4$
Alternate Hypothesis: $\mu A10 < \mu A4$

Where µA=mean pain reduction in Ampion™ arm; µP=mean pain reduction in placebo arm. Lower mean change scores indicate greater pain reduction.

10.3.6 Definition of Study Visits

This clinical trial has a total of 9 study visits, including telephone contacts, during the 12 week study (see Table 6.2). The time-on-study for each patient observation will be defined relative to Day 1, the day of the initial dose. For analysis, the Baseline measure is the latest measure prior to initiation of treatment.

10.3.7 Number of patients to receive study drug

Planned enrollment is 80 patients in each treatment arm, for a total sample population of 320 patients. Assuming a 5% dropout rate, a total of 76 patients will be enrolled in each of 4 treatment arms, for a total of 304 patients.

10.3.8 Disposition of patients

Disposition of patients, including study completion status and response to therapy as measured by WOMAC pain subscores, will be summarized by treatment group (Ampion™ and placebo), volume (10ml and 4ml), age group, race and gender for each of the analysis populations.

10.3.9 Interim analysis

There will be no interim analysis.

10.3.10 Blinding and randomization

Patients will be assigned to treatment by a randomization schedule generated by an independent statistician. Ampion™ and placebo will be provided in blinded study vials labeled with the appropriate information and packed into patient kits. Each patient kit will contain labeled vials, blinded for drug content, study syringes for either a 4mL or 10mL dose and needles that are 20 gauge and 1.5 inches long. Patient kits will be labeled with double-panel labels, blinded for drug content.

The Sponsor, the investigator, and all study staff having a role in the day-to-day conduct of the Study will remain blinded to treatment. Where required, safety personnel may be unblinded to a particular patient's treatment assignment to meet reporting requirements to Regulators. Unblinding may occur by unmasking the double-panel label that contains the treatment disclosure panel.

A final Statistical Analysis Plan will be issued prior to unblinding of data to adjust for any changes to the protocol or unexpected issues in study conduct and data that affect the planned analyses.

10.3.11 Data presentation

10.3.11.1 Demographic and Baseline Characteristics:

Demographic (e.g., age, sex, race, ethnicity) and baseline characteristics (e.g., weight, height) summarized using descriptive statistics, overall and by treatment group for the ITT analysis population.

10.3.11.2 Medical History and Physical Examination:

The number and percent of patients with past and current medical disorders at the time of randomization will be presented overall and by treatment group for the ITT analysis population. Results of any abnormalities documented from the abbreviated physical examination at Baseline and Week 12, will be summarized overall and by treatment group for the safety and ITT analysis populations.

10.3.11.3 Concomitant Medications or Treatments:

The number and percent of patients receiving concomitant medications or treatments prior to and during the study and at the final visit will be tabulated and presented overall and by treatment group for the ITT analysis population. Concomitant medications/treatments will be summarized using descriptive statistics and will be presented by type of drug (WHO DRUG classification) overall and by treatment group for the safety and ITT analysis populations.

10.3.11.4 Safety data:

Safety data will be evaluated by changes in vital sign measurements and the frequency and severity of AEs. Concomitant medication will be recorded for safety.

Adverse events:

The Investigator is responsible for monitoring the safety of patients who have enrolled in the study. All AEs considered to be possibly related to Ampion™ will be followed until the event resolves or stabilized without further change. Patients will be followed for the occurrence of AEs until 12 weeks after the first dose of study medication.

Investigators are required to document all AEs occurring during the clinical trial, commencing with the first day of treatment and including the protocol-defined post-treatment follow-up period on the appropriate CRF pages.

The severity of AEs (mild, moderate, severe), relatedness (related, possibly related, unrelated) along with the duration, action taken, and outcome (e.g., study withdrawal) will also be recorded. In addition, events meeting the criteria of a Serious Adverse Event (SAE) must be reported to the Sponsor within 24 hours on the SAE reporting forms.

10.3.11.5 Efficacy data:

All efficacy variables will be assessed at Baseline (Day 1), Week 2 (Day 14 ± 5), Week 4 (Day 30 ± 5), Week 6 (Day 42 ± 5), Week 8 (Day 56 ± 5), Week 10 (Day 70 ± 5) and Week 12 (Day 84 ± 5). Except where noted to be otherwise, all statistical tests will be two-sided and will be at the 5% level of significance.

Unless otherwise specified, continuous variables will be summarized with the number of non-missing observations, mean, standard deviation, median, minimum, and maximum displayed. Categorical data will be summarized as counts and percentages. All efficacy assessments will be summarized as the measured value and as the change from baseline by treatment at each timepoint. Summary statistics will include number of observations, mean, standard deviation, median, minimum and maximum. Change from baseline will also include a 95% confidence interval.

Except where otherwise specified, missing data will not be estimated or carried forward in any of the descriptive analyses. No multiple comparison adjustment will be made for the secondary efficacy analyses. Data transformation or use of rank-based tests may be used if endpoints depart substantially from a normal distribution.

Baseline is defined as the last pre-treatment assessment. Because the secondary analyses are considered supportive to the primary analysis (i.e., not required to demonstrate efficacy of the test article), there is no requirement under ICH to adjust for multiplicity.

10.4 STUDY ENDPOINTS

10.4.1 Primary Endpoint

- Change in the Western Ontario and McMaster Universities (WOMAC) Osteoarthritis Index 3.1 pain subscore by 5-point Likert scale between Baseline and Week 12.

The primary variable is the average score of the five WOMAC A (pain) subscale questions:

In the last 12 hours how much pain have you had in the study knee:

1. When walking on a flat surface?
2. When going up or down stairs?
3. At night while in bed? (that is - pain that disturbs your sleep)
4. While sitting or lying down?
5. While standing?

The primary analysis of the difference between treatment groups in the mean change in WOMAC A pain subscore from Baseline to Week 12 will use an analysis of covariance model, adjusted for baseline WOMAC A pain subscore.

10.4.2 Secondary Endpoints

- Change in WOMAC A pain subscore between baseline and Weeks 2, 4, 6, 8, and 10 Change in WOMAC B stiffness subscore between baseline and Weeks 2, 4, 6, 8, 10 and 12
- Change in WOMAC C physical function subscore between baseline and Weeks 2, 4, 6, 8, 10 and 12
- Change in PGA between baseline and Weeks 6, 8, 10 and 12
- Response status based on the OMERACT-OARSI criteria at Weeks 2, 4, 6, 8, 10 and 12
- Change in WOMAC A pain subscore questions 1 and 2 (pain with movement) between baseline and Weeks 2, 4, 6, 8, 10 and 12
- Change in WOMAC A pain subscore questions 3−5 (resting pain) between baseline and Weeks 2, 4, 6, 8, 10 and 12
- Use of rescue analgesia (amount of acetaminophen used)
- Incidence and severity of treatment-emergent adverse events (TEAEs).

Secondary efficacy analyses include the difference between treatment groups in the mean change in WOMAC B, WOMAC C, WOMAC A1-2, WOMAC A3-5, PGA and amount of acetaminophen used from baseline to Week 12 using an analysis of covariance model, adjusted for baseline value.

Percent responders using the OMERACT-OARSI criteria will be analyzed using separate logistic regression models for each visit, adjusted for baseline WOMAC A pain subscore.

10.4.3 Exploratory Secondary Endpoints

Approximately 20 subjects injected with 10 mL saline or Ampion™, randomized 1:1, will be evaluated using the WORMS system. Change in the total WORMS score will be summarized between screening and week 12. All WORMS subscores will be summarized by treatment, including cartilage morphology and cartilage signal, at the two time points as well as their change. The significance of the change at week 12 will be tested against the null hypothesis of no change using a paired t-test. The difference between groups in the mean change at week 12 will be tested against the null hypothesis of no difference using an analysis of covariance model, adjusted for baseline value.

10.5 MISSING AND SPURIOUS DATA

All data collected under this study protocol will be included in the assessment of patient safety. Missing or incomplete AE data will assume greatest relationship to study drug and/or severity.

For the two primary effectiveness endpoint analyses (WOMAC pain change) in the volume by study drug analysis and in the repeat injection by study drug analysis, missing change scores will be imputed in when the ITT analysis population is used. The primary approach to imputing missing data will be by multiple imputations (SAS PROC MI and SAS PROC MIANALYZE). A sensitivity analysis will be conducted in which the missing 12-week endpoint is replaced by the baseline WOMAC pain score.

11 REGULATORY, ETHICAL AND LEGAL OBLIGATIONS

11.1 DECLARATION OF HELSINKI

The Principal Investigator will ensure that this Study is conducted in accordance with the most recent revision of the Declaration of Helsinki.

11.2 GOOD CLINICAL PRACTICE

The Study will be conducted according to the study protocol and to Standard Operating Procedures (SOPs) that meet the guidelines provided by the International Conference on Harmonisation (ICH) for Good Clinical Practice in clinical studies.

11.3 INSTITUTIONAL REVIEW BOARDS/ETHICS COMMITTEES

Before implementing this study, the protocol, the proposed patient informed consent forms and other information for the patients, must be reviewed by a properly constituted committee or committees responsible for approving clinical studies. The IRB/IEC written, signed approval letter/form must contain approval of the designated investigator, the protocol (identifying protocol title, date and version number), and of the patient informed consent form (date, version).

Any change or addition to the protocol can only be made in a written protocol amendment that must be approved by the Sponsor, the IRB/IEC and the Health Authorities.

11.4 REGULATORY AUTHORITY APPROVAL

Before this study is implemented, the protocol must be approved by the relevant regulatory authority.

11.5 INFORMED CONSENT

The investigator must fully inform the patient of all pertinent aspects of the trial including the written information approved/favorably assessed by the IRB/IEC.

Prior to the start of the pre-study examination, the written informed consent form must be signed and personally dated by the patient and by the physician who conducted the informed consent discussion. One copy of the written information and signed consent form must be given to each patient and 1 copy must be retained in the investigator's study records.

11.6 PATIENT CONFIDENTIALITY AND DISCLOSURE

Data on patients collected on eCRFs during the trial will be documented in an anonymous fashion and the patient will only be identified by the patient number, and by his/her initials If, as an exception, it is necessary for safety or regulatory reasons to identify the patient, all parties are bound to keep this information confidential.

The investigator will guarantee that all persons involved will respect the confidentiality of any information concerning the trial patients. All parties involved in the study will maintain strict confidentiality to assure that neither the person nor the family privacy of a patient participating in the trial is violated. Likewise, the appropriate measures shall be taken to prevent access of non-authorized persons to the trial data.

11.7 COLLECTION, MONITORING AND AUDITING STUDY DOCUMENTATION, AND DATA STORAGE

11.7.1 Collection of Data and Monitoring Procedures

This study will use a 21 CFR Part 11 compliant electronic data capture system (eDC). An electronic case report form (eCRF) is used for data recording. All data requested on the eCRF must be entered and all missing data must be accounted for.

The data will be checked for completeness and correctness as it is entered by the real-time online checks applied by the eDC system. Off-line checks will also be run to perform any additional data review required. Discrepancy reports will be generated accordingly and transferred to the study center for resolution by the investigator or his/her designee.

Accurate and reliable data collection will be assured by verification and cross–check of the eCRF against the investigator's records by the study monitor (source document verification), and the maintenance of a study drug–dispensing log by the investigator.

Before study initiation, at a site initiation visit or at an investigator's meeting, a Sponsor representative will review the protocol and case report forms with the investigators and their staff. During the study a monitor will visit the site regularly to check the completeness of patient records, the accuracy of entries on the case report forms, the adherence to the protocol and to Good Clinical Practice, the progress of enrollment. The monitor will ensure during on-site visits that study medication is being stored, dispensed and accounted for according to specifications. Key trial personnel must be available to assist the monitors during these visits.

The investigator must give the monitor access to relevant hospital or clinical records, to confirm their consistency with the case report form entries. No information in these records about the identity of the patients will leave the study center. Monitoring standards require full verification for the presence of informed consent, adherence to the inclusion/exclusion criteria, documentation of SAEs and the recording of primary efficacy and safety variables. Additional checks of the consistency of the source data with the eCRFs are performed according to the study-specific monitoring plan.

11.7.2 Auditing Procedure

In addition to the routine monitoring procedures the Sponsor or the regulatory authority can conduct an audit or an inspection (during the study or after its completion) to evaluate compliance with the protocol and the principles of Good Clinical Practice.

The investigator agrees that representatives of the Sponsor and Regulatory Authorities will have direct access, both during and after the course of this study, to audit and review all study-relevant medical records.

11.7.3 Retention of Documents

The investigator must maintain source documents for each patient in the study, consisting of all demographic and medical information, including laboratory data, MRI etc, and keep a copy of the signed informed consent form. All information on case report forms must be traceable to these source documents in the patient's file. Data without a written or electronic record will be defined before trial start and will be recorded directly on the case report forms, which will be documented as being the source data.

11.8 DISCLOSURE OF INFORMATION

All information provided to the investigator by Ampio Pharmaceuticals, Inc. or their designee, will be kept strictly confidential. No disclosure shall be made except in accordance with a right of publication granted to the investigator.

No information about this study or its progress will be provided to anyone not involved in the study other than to Ampio Pharmaceuticals, Inc or its authorized representatives, or in confidence to the IRB, or similar committee, except if required by law.

11.9 DISCONTINUATION OF THE STUDY

It is agreed that, for reasonable cause, either the investigator or Ampio Pharmaceuticals, Inc., may terminate the investigator’s participation in this study after submission of a written notice. Ampio Pharmaceuticals, Inc., may terminate the study at any time upon immediate notice for any reason, including the Sponsor’s belief that discontinuation of the study is necessary for the safety of patients.

11.10 STUDY REPORT, PUBLICATION POLICY AND ARCHIVING OF STUDY DOCUMENTATION

11.10.1 Study Report and Publication Policy

An ICH-compliant integrated clinical and statistical report will be prepared upon completion of the study and data analysis. The results of the study will be published in a relevant peer-reviewed journal, with authorship status and ranking designated according to the acknowledged contributions of participating investigators, institutions and the Sponsor.

11.10.2 Study Documents

The investigator must maintain source documents for each patient in the study, consisting of all demographic and medical information, questionnaires, including laboratory data, MRI (as applicable), etc., and keep a copy of the signed informed consent form. All information on the e-case report forms must be traceable to these source documents in the patient's file. Data without a written or electronic record will be defined before trial start and will be recorded directly on the e-case report forms, which will be documented as being the source data.

11.10.3 Archiving of Documents

Essential documents, as listed below, must be retained by the investigator for as long as needed to comply with national and international regulations. The Sponsor will notify the investigator(s)/institution(s) when the study-related records are no longer required. The investigator agrees to adhere to the document retention procedures by signing the protocol. Essential documents include:

- IRB/IEC/REB approvals for the study protocol and all amendments
- All source documents and laboratory records
- CRF copies (electronic copies on a CDROM)
- Patients' informed consent forms (with study number and title of trial)
- FDA form 1572
- Any other pertinent study document.

12 REFERENCES

1. Bar-Or D, Bar-Or R, Rael LT, et al. Heterogeneity and oxidation status of commercial human albumin preparations in clinical use. *Crit Care Med*. 2005;33:1638-41.

2. Bar-Or D, Thomas GW, Bar-Or R, et al. Commercial human albumin preparations for clinical use are immunosuppressive in vitro. *Crit Care Med*. 2006;34:1707-12.

3. Bellamy N, Buchanan WW, Goldsmith CH et al. Validation study of WOMAC: a health status instrument for measuring clinically important patient relevant outcomes to antirheumatic drug therapy in patients with osteoarthritis of the hip or knee. *J Rheumatol*. 1988 Dec;15(12):1833-40.

4. Dillon CF, Rasch EK, Gu Q, Hirsch R. Prevalence of knee osteoarthritis in the United States: arthritis data from the Third National Health and Nutrition Examination Survey 1991-94. *J Rheumatol*. 2006 Nov;33(11):2271-9.

5. Evans TW. Review article: albumin as a drug – biological effects of albumin unrelated to oncotic pressure. *Aliment Pharmacol Ther*. 2002;16 (Suppl. 5); 6-11.

6. Felson DT, Anderson JJ, Naimark A, et al. Obesity and knee osteoarthritis: The Framingham study. *Ann Int Med*. 1988;109:18-24.

7. Guccione AA. Arthritis and the process of disablement. *Phys Ther*. 1994 May;74(5):408-14.

8. Mallinckrodt CH, Clark WS, David SR. Accounting for dropout bias using mixed-effects models. *J Biopharm Stat*. 2001 Feb-May;11(1-2):9-21.

9. Navarro-Sarabia F, Coronel P, Collantes E, Navarro FJ, de la Serna AR, Naranjo A, Gimeno M, Herrero-Beaumont G; AMELIA study group. A 40-month multicentre, randomized placebo-controlled study to assess the efficacy and carry-over effect of repeated intra-articular injections of hyaluronic acid in knee osteoarthritis: the AMELIA project. *Ann Rheum Dis*. 2011;70:1957-62.

10. Peterfy C.G., M.D. Ph.D., Guermazi A M.D., S. Zaim M.D., P. F. J. Tirman M.D., Y. Miaux M.D., D. White Ph.D., M. Kothari Ph.D., Y. Lu Ph.D., K. Fye M.D., S. Zhao Ph.D. H. K. Genant M.D. Whole-Organ Magnetic Resonance Imaging Score (WORMS) of the knee. OsteoArthritis and Cartilage (2004) 12, 177–190.

11. Quinlan GJ, Martin GS, Evans TW. Albumin: biochemical properties and therapeutic potential. *Hepatology*. 2005;41:1211-1219.

12. Shimonkevitz R, Thomas GW, Slone DS, et al. A diketopiperazine fragment of human serum albumin modulates T-lymphocyte cytokine production through Rap1. *J Trauma*. 2008;64:35-41.

13. Vincent JL, Navickis RJ, Wilkes MM. Morbidity in hospitalized patients receiving human albumin: A meta-analysis of randomized, controlled trials* *Crit Care Med*. 2004;32:2029–38.

14. Zhang W, Nuki G, Moskowitz R.W. et al. OARSI recommendations for the management of hip and knee osteoarthritis Part III: changes in evidence following systematic cumulative update of research published through January 2009. *Osteoarthritis and Cartilage.* 2010;18:476-499.

13 APPENDICES

13.1 CONTACT LIST

SPONSOR:
Ampio Pharmaceuticals, Inc.
5445 DTC Parkway Suite 925
Greenwood Village, CO 80111
720-437-6500

PRIMARY SPONSOR CONTACT:
Vaughan Clift, MD
5445 DTC Parkway Suite 925
Greenwood Village, CO 80111
720-437-6500
vclift@ampiopharma.com

SECONDARY SPONSOR CONTACT:
Holli Loose, MSc
5445 DTC Parkway Suite 925
Greenwood Village, CO 80111
720-437-6500
hloose@ampiopharma.com

STUDY MONITOR:
Terrie Hopton
3100 Bristol Street, Suite 250
Costa Mesa, CA 92626
714-460-7363
thopton@promedica-intl.com
