# Supplementary material for: A Randomized Clinical Trial to Evaluate Two Doses of an Intra-Articular Injection of LMWF-5A in Adults with Pain Due to Osteoarthritis of the Knee
Source: PLoS One. 2014 Feb 3;9(2):e87910. doi: 10.1371/journal.pone.0087910 (PMC3912151; doi:10.1371/journal.pone.0087910)
Supplement: SAP S1 — Statistical Analysis Plan AP-003-A (Version 2.0, 8 August 2013). (PDF) [file pone.0087910.s002.pdf]

STATISTICAL ANALYSIS PLAN APPROVAL SHEET

Protocol Number: AP-003-A

Product: Ampion™

Protocol Title: A Randomized, Placebo-Controlled, Double-Blind Study to Evaluate the Efficacy and Safety of Two Doses of Intra-Articular Injection of Ampion™ in Adults With Pain Due to Osteoarthritis of the Knee

Author: Gerard Smits, Ph.D.

Version: 2.00

Version date: August 8, 2013

Approved by:

[signature] 8 Aug 2013

Name Date

[signature] 08aug2013

Name Date

Name Date

STATISTICAL ANALYSIS PLAN APPROVAL SHEET

Protocol Number: AP-003-A

Product: Ampion™

Protocol Title: A Randomized, Placebo-Controlled, Double-Blind Study to Evaluate the Efficacy and Safety of Two Doses of Intra-Articular Injection of Ampion™ in Adults With Pain Due to Osteoarthritis of the Knee

Author: Gerard Smits, Ph.D.

Version: 2.00

Version date: August 8, 2013

Approved by:

[signature] 8/8/2013

Name Date

Name Date

Name Date

STATISTICAL ANALYSIS PLAN

Phase 2, *A Randomized, Placebo-Controlled, Double-Blind Study to Evaluate the Efficacy and Safety of Two Doses of Intra-Articular Injection of Ampion™ in Adults With Pain Due to Osteoarthritis of the Knee*

Protocol Number AP-003-A

**Version:**	2.00
**Date:**	08 August 2013
**Biostatistician:**	Gerard Smits, Ph.D.

Table of Contents

Version date: July 17, 2013 1
Version: 1.00 2

1. INTRODUCTION 5

2. OBJECTIVES 5
   2.1 Primary 5
   2.2 Secondary 5
   2.3 Study Design 5
   2.4 Sample Size 6

3. STUDY ENDPOINTS AND COVARIATES 6
   3.1 Primary Endpoint 6
   3.2 Secondary Endpoints 7
   3.3 Exploratory Endpoints 7

4. HYPOTHESES 8

5. DEFINITIONS 8
   5.1 Abbreviations 8
   5.2 Definitions 9

6. ANALYSIS SUBSETS 11
   6.1 Data Subsets 11
      6.1.1 Safety Analysis Set 11
      6.1.2 Intent to Treat (ITT) Analysis Set 11
      6.1.3 Per Protocol (PP) Analysis Set 11
      6.1.4 Subset Analysis Set 12

7. INTERIM ANALYSIS AND EARLY STOPPING GUIDELINES 12
   7.1 Data Handling and Electronic Transfer of Data 12
   7.2 Handling of Missing and Incomplete Data 12
   7.3 Detection of Bias 12
   7.4 Outliers 12
   7.5 Testing/Validation Plan 12

8. STATISTICAL METHODS OF ANALYSIS 12
   8.1 General Principles 13
   8.2 Subject Accountability 13
   8.3 Demographic and Baseline Characteristics 13
   8.4 Safety Analyses 13
      8.4.1 Concomitant Medications 13
      8.4.2 Clinical Laboratory Tests 14
      8.4.3 Vital Signs 14
      8.4.4 Physical Examinations 14

8.5 Efficacy Analyses 14
8.5.1 Primary Effectiveness Endpoint 15
8.5.2 Secondary Effectiveness Endpoints 15
8.5.3 Exploratory Analysis 16
8.5.4 Multiplicity Adjustment 17
8.5.5 Post Hoc/Ad Hoc Analyses 17

9. CHANGES FROM PROTOCOL-SPECIFIED ANALYSES 17

10. LIST OF PLANNED TABLES, FIGURES, AND LISTINGS 17
10.1 Tables 17
10.2 Listings 23
10.3 Figures 24

11. LITERATURE CITATIONS / REFERENCES 24

12. HANDLING OF MISSING OR INCOMPLETE DATES FOR ADVERSE EVENTS AND CONCOMITANT MEDICATIONS 24
12.1 Imputation Rules for Partial or Missing Stop Dates 24

13. Schedule of Assessments and Procedures 26

1. INTRODUCTION

This statistical analysis plan (SAP) outlines the proposed statistical methods to be implemented during the review of data to ensure that it confirms with categories determined by the CRF or the anticipated ranges for continuous variables and analysis of data collected within the scope of Ampion Protocol AP-003-A, "A Randomized, Placebo-Controlled, Double-Blind Study to Evaluate the Efficacy and Safety of Two Doses of Intra-Articular Injection of Ampion™ in Adults With Pain Due to Osteoarthritis of the Knee," dated 15 May, 2013.

It is not intended that each and every table, listing, or graph will be included in the clinical study report (CSR). It is also possible that additional analyses will be conducted after review of the data. Any analyses or summaries not specified in the SAP, but performed after review of the data, will be identified in the CSR as post hoc.

2. OBJECTIVES

2.1 Primary

The primary trial objectives are to evaluate the greater efficacy of Ampion™ versus placebo and to evaluate the greater efficacy of 10 mL Ampion™ versus 4 mL Ampion™ in improving knee pain, when applied to patients suffering from OA of the knee.

2.2 Secondary

The secondary trial objectives include: evaluation of the safety of an intra-articular injection of Ampion™ when applied to patients suffering from OA of the knee, evaluation of the efficacy of intra-articular injection of Ampion™ and placebo on stiffness and function when applied to patients suffering from OA of the knee and evaluation of responder status defined by the Outcome Measures in Rheumatology Clinical Trials and Osteoarthritis Research Society International (OMERACT-OARSI) criteria.

Exploratory objectives are to: analyze the effect, if any, of Ampion™ versus saline on intra-articular inflammatory growth markers in a subset of patients and to assess any radiological changes visible in MRI. It is of interest also to explore the presence of subsets of patients, defined on the basis of baseline conditions that respond differently to treatment.

2.3 Study Design

This is a randomized, placebo-controlled, double-blind study with a 28 day screening period for each patient followed by a 12 week participation period. A total of 320 patients, 80 patients per study arm, with OA knee pain will be randomized 1:1:1:1 across 4 study arms: 4mL Ampion™, 4mL placebo, 10mL Ampion™ or 10mL placebo. A subset of patients, approximately 20 total patients, at one site randomized 1:1 from the

10mL injection arms will undergo a 1-2mL aspiration of the index knee (Baseline, Weeks 6 and 12) and MRI (Baseline and Week 12).

The clinical effects of treatment on OA pain will be evaluated during clinic visits at 6 and 12 weeks and telephone contacts at 2, 4, 8 and 10 weeks, using the Western Ontario and McMaster Universities Arthritis Index (WOMAC®) osteoarthritis Index 3.1, and the Patient's Global Assessment of disease severity (PGA). The WOMAC® is a validated pain scoring system and sets the standard for the patient response. In order not to bias the collection of data, only questions from the validated WOMAC pain scale will be asked of patients. Clinical meaningfulness will be determined by the end results of this trial, specifically by the apparent clinical benefit versus any adverse events or any increased apparent risk.

Safety will be assessed by recording adverse events (through 24 hours post-dose and at all follow-up contacts), physical examination and vital signs (Baseline, Weeks 6, and 12), and clinical laboratory tests for the 10mL study arms (Baseline, Weeks 6, and 12).

2.4 Sample Size

A total of approximately 320 patients will be enrolled in the study using a 1:1:1:1 ratio across the four study arms (4mL placebo, 4mL Ampion™, 10mL placebo, 10mL Ampion™). 80 total patients will be randomized into each study arm to achieve the required number of 75 patients per treatment arm to achieve 80% power.

Phase 2 work completed by Ampio, indicates that the standardized treatment effect at 12 weeks, relative to placebo, is approximately 0.43 (effect: 1.23, SD 2.88) using an 11-point National Institutes for Health numerical rating scale for pain. For the analysis of, saline v Ampion™ (by 4mL v 10mL) treatment effects assumed are a SD of 0.9 for the WOMAC A pain score with mean pain decreases of 2.0 for 10mL Ampion™, 1.5 for 4mL Ampion™, and 1.0 for both saline placebo arms. With 75 evaluable in each of 4 arms, the power to demonstrate both main effects or an interaction between the two main effects is greater than 80% using 2-tailed alpha of 0.05.

3. STUDY ENDPOINTS AND COVARIATES

3.1 Primary Endpoint

The primary effectiveness endpoint is the change in the Western Ontario and McMaster Universities (WOMAC) pain subscore by 5-point Likert scale between Baseline and Week 12.

The primary endpoint is the average score of the five WOMAC A (pain) subscale questions:

In the last 12 hours how much pain have you had in the study knee:

1. when walking on a flat surface?
2. when going up or down stairs?
3. at night while in bed? (that is - pain that disturbs your sleep)
4. while sitting or lying down?
5. while standing?

3.2 Secondary Endpoints

- Change in WOMAC A pain subscore between baseline and Weeks 2, 4, 6, 8, and 10
- Change in WOMAC B stiffness subscore between baseline and Weeks 2, 4, 6, 8, 10, and 12
- Change in WOMAC C physical function subscore between baseline and Weeks 2, 4, 6, 8, 10, and 12
- Change in PGA between baseline and Weeks 6, 8, 10, and 12
- Response status based on the OMERACT-OARSI criteria at Weeks 2, 4, 6, 8, 10, and 12
- Change in WOMAC A pain subscore average of questions 1 and 2 (pain with movement) between baseline and Weeks 2, 4, 6, 8, 10, and 12
- Change in WOMAC A pain subscore average of questions 3–5 (resting pain) between baseline and Weeks 2, 4, 6, 8, 10, and 12
- Use of rescue analgesia (amount of acetaminophen used)
- Incidence and severity of treatment-emergent adverse events (TEAEs)

3.3 Exploratory Endpoints

- For the subset of subjects tested by MRI and biomarkers, exploratory endpoints include change in the WORMS subscores between baseline/randomization and week 12 with a focus on medial femoro-tibial (MFT) and lateral femoror-tibial (LFT). Subscores include cartilage morphology and signal, bone marrow edema, and Synovitis/Effusion. Subscore values are generated by summing the component values.
- For the subset of subjects tested by MRI and given additional laboratory testing, biomarkers serum CS-846, Serum PIIANP, Serum COMP, and Serum CM2 will be summarized by treatment arm and change from baseline determined.
- Due to the expected interrelationship between the WOMAC Pain, Function, and Stiffness scores (e.g., subsequent to an increase in activity, associated with improved function scores, an increase in pain may result), the sequential

pattern and interrelationship between the three WOMAC measures will be explored.

4. HYPOTHESES

Reduction in Pain, as measured by the WOMAC A pain subscore by 5-point Likert scale between baseline and Week 12 will be greater in patients treated with Ampion™ than with saline placebo:

Null Hypothesis: $\mu A = \mu P$
Alternate Hypothesis: $\mu A < \mu P$

Reduction in Pain, as measured by the WOMAC A pain subscore by 5-point Likert scale between baseline and Week 12 will be greater in patients treated with Ampion™ 10mL than with Ampion™4mL:

Null Hypothesis: $\mu A10 = \mu A4$
Alternate Hypothesis: $\mu A10 < \mu A4$

Where μA=mean pain reduction in Ampion™ arm; μP=mean pain reduction in placebo arm. Lower mean change scores indicate greater pain reduction.

5. DEFINITIONS

5.1 Abbreviations

Abbreviation	Definition
ANCOVA	Analysis of Covariance
AR (1)	First-order autoregressive
ATC	Anatomical Therapeutic Chemical Classification System
LFT	Lateral femoro-tibial
LOCF	Last observation carried forward
MedDRA®	Medical Dictionary for Regulatory Activities
MFT	Medial femoro-tibial
mg	Milligram
mL	Milliliter
mmHg	Millimeters of mercury
NA	Not applicable
ng	Nanogram

NSAID	Non-steroidal anti-inflammatory drug
OA	Osteoarthritis
OMERACT-OARSI	Outcome measures in rheumatology clinical trials and osteoarthritis research society international
PGA	Patient's global assessment of disease severity
PP	Per protocol population
qd	Once daily
RBC	Red blood cell
REB	Research Ethics Board
SAE	Serious adverse event
SAS	Statistical Software from SAS Institute
SD	Standard deviation
SEM	Standard error of the mean
SMC	Safety Monitoring Committee
SOP	Standard operation procedure
TEAE	Treatment-emergent adverse event
WBC	White blood cell
WCC	White cell count
WO	Washout
WOMAC	Western Ontario and McMaster Universities Arthritis Index
WORMS	Whole-Organ Magnetic Resonance Imaging Score

5.2 Definitions

Adverse Event (AE)

An adverse event (AE) is defined as any undesired medical occurrence in a patient or clinical investigation patient receiving a pharmaceutical product and which does not necessarily have a causal relationship with this treatment. An AE can therefore be any unfavorable sign and unintended sign (including an abnormal laboratory finding), symptom, or disease temporarily associated with the use of a study drug, whether or not related to the study drug.

AEs will be graded for severity using the following categories. Missing grade will be assigned a grade of 3 (severe) in tabulations.

Grade 1 (MILD): The symptom is barely noticeable to the study patient and does not influence performance or functioning. Concomitant medication is not ordinarily indicated for relief of mild AEs.

Grade 2 (MODERATE): The symptom is of sufficient severity to make the study patient uncomfortable and to influence performance of daily activities. Concomitant medication may be indicated for relief of moderate AEs.

Grade 3 (SEVERE):_The symptom causes severe discomfort, sometimes of such severity that the study patient cannot continue in the study. Daily activities are significantly impaired or prevented by the symptom. Concomitant medication may be indicated for relief of severe AEs.

Relationship to study drug will be codes using the following categories. Missing relatedness will be assigned to related in tabulations.

Unrelated: The adverse event is unlikely to have been caused by study drug.
Possibly related: It is unclear whether the adverse event may have been caused by study drug.
Related: The adverse event is likely to have been caused by study drug.

Serious Adverse Event:

A serious adverse event (SAE) is defined as an adverse event that

- Results in death
- Is life-threatening (patient is at immediate risk of death from the event as it occurred)
- Requires in-patient hospitalization (formal admission to a hospital for medical reasons) or prolongation of existing hospitalization
- Results in persistent or significant disability/incapacity
- Results in a congenital anomaly/birth defect

Treatment-Emergent AE:

A treatment-emergent AE (TEAE) is any AE that begins or increases in severity after the initial dose of study drug.

Age

Subject's age is defined as its integer value in years at enrollment.

Baseline

For any variable, unless otherwise defined, baseline is the last assessment taken prior to the first study drug administration.

Change from Baseline:
The arithmetic difference between a post-baseline value and the baseline value:
Change from Baseline = (Post-baseline Value – Baseline Value)
Percentage Change from Baseline = [(Post-baseline Value – Baseline Value) / Baseline Value] x 100

End of Study

End of study is at Visit 9 (day 84 ± 7 days), unless terminated early.

Enrollment date

Enrollment date is the same as the randomization date and is designated Day 0.

Study drug

Study drug in this study is Ampion or placebo.

Randomization date

Randomization date is the day the subject is assigned a randomization number on study Day 0.

Study Day 0

Day 0 is defined as the first day that study drug is administered to the subject.

Study Day

Day of treatment: study day = (visit date - date of Study Day 0)

OMERACT-OARSI Response

A response is defined as 1) a 50% decrease or a 1.0 unit drop from baseline in WOMAC pain score; or 2) at least two of the following conditions being met: a) a 20% decrease in WOMAC pain or a 0.5 unit drop; b) a 20% decrease in WOMAC difficulty score or a 0.5 unit drop; or c) a 20% decrease in PGA or a 0.5 unit drop.

6. ANALYSIS SUBSETS

6.1 Data Subsets

6.1.1 Safety Analysis Set

The safety analysis population is defined as all patients who are randomized and receive study medication (Ampion™ or placebo at 4mL or 10mL). Patients will be analyzed as treated. Summaries of data will include all data assigned to a nominal visit whether within the visit window.

6.1.2 Intent to Treat (ITT) Analysis Set

The intent-to-treat (ITT) analysis population is defined as all patients who are randomized, receive study medication (Ampion™ or placebo at 4mL or 10mL) and have at least one post-baseline observation. All efficacy analyses will be performed in the ITT population. Patients will be analyzed as randomized.

6.1.3 Per Protocol (PP) Analysis Set

The per protocol analysis population is defined as all patients included in the ITT analysis who met all entry criteria and had no major protocol violations. All efficacy analyses will be repeated in the per-protocol population. These analyses will be supportive of the ITT

analysis. Patients will be analyzed as treated. Summaries of and analyses of data will exclude all data outside of the visit window.

6.1.4 Subset Analysis Set

A subset of 20 subjects injected with 10 mL will have a Whole-Organ Magnetic Resonance Imaging Score (WORMS) measured at baseline and Week 12. These subjects will also have additional laboratory measures (biomarkers: serum CS-846, Serum PIIANP, Serum COMP, and Serum CM2) recorded.

7. INTERIM ANALYSIS AND EARLY STOPPING GUIDELINES

No formal interim analysis is planned. The study may be stopped upon recommendation by the safety monitoring committee.

7.1 Data Handling and Electronic Transfer of Data

See Data Management Plan (DMP).

7.2 Handling of Missing and Incomplete Data

Incomplete adverse event and concomitant medication dates will be imputed as described in Section 12. If imputed dates are used, then they will be identified as such in the final study report. If an AE start date is missing or partially missing and no additional information is available from the site in order to establish whether the event started before or after the dose of study drug, the event will be considered to have started after dose. Partially missing dates where the month and year is prior to Day 0 will not be classified as post dose.

7.3 Detection of Bias

Any breaking of the blind for individual subjects prior to formal unblinding will be documented in the clinical study report. Data collected after unblinding will be noted.

7.4 Outliers

No formal outlier tests are planned. Values that are outside the pre-defined range, such as a WOMAC sore not between zero and four, or a PGA score not between one and five, would be queried and excluded if necessary prior to database lock.

7.5 Testing/Validation Plan

All statistical analyses will be programmed using SAS® software version 9.2, or later. Graphic displays may be produced using R, version 3.0.0, or later. Standard macros will be used in programming when possible. Testing and validation plans for all programs will be developed in accordance with contract research organization guidelines and will include independent programming of tables and analyses.

8. STATISTICAL METHODS OF ANALYSIS

8.1 General Principles

Data will be summarized by each treatment arm and by pooled Placebo and Ampion™ arms. Descriptive statistics on continuous variables will include mean, standard deviation, median, 25th and 75th percentiles, and range. Change from baseline will include a 95% confidence interval. Categorical variables will be summarized using frequency counts and percentages. Data listings of individual subject's data will be provided. Statistical testing will be performed using a two-tailed alpha of 0.05, unless otherwise specified.

8.2 Subject Accountability

The number of subjects who are randomized, receive study drug, and complete the study will be summarized. The number of subjects included in the safety, ITT, and PP analysis sets will be included in the table. Attendance at each visit, including missed visits, discontinuations, lost to follow-up, and percentage accountability will be summarized. A list of subjects who withdraw early will be provided. It will include the reason and timing of the withdrawal. Similarly, the reason any subject is excluded from an analysis set will also be provided. In addition, significant known protocol deviations will be noted for individual subjects; a summary table may also be provided.

8.3 Demographic and Baseline Characteristics

Age, race, ethnicity, sex, height, weight, body mass index, history or prior study knee injections, and Kellgren Lawrence grade will be summarized by treatment arm for all subjects receiving study drug, using descriptive statistics. Distribution of data across the 4 treatment arms will be assessed by Fisher's exact tests form binary endpoints, Pearson Chi-square tests for categorical variables, and by Kruskal-Wallis rank-based tests for continuous endpoints.

8.4 Safety Analyses

The safety profile will be based on adverse events, concomitant medications, vital signs, physical examinations, and clinical laboratory measurements. All treated subjects will be included in the safety analyses

Adverse Events

Adverse events will be grouped by system organ class and by preferred term within system organ class according to the latest version of the MedDRA coding dictionary. The number of subjects reporting at least 1 adverse event and each adverse event will be summarized treatment group. Tables and/or narratives of any on-study death, serious or significant adverse events, including early withdrawals because of adverse events, will be provided should they occur.

8.4.1 Concomitant Medications

The number and percent of patients receiving concomitant medications or treatments prior to and during the study and at the final visit will be tabulated and presented overall

and by treatment group for the Safety analysis dataset. Concomitant medications and treatments will be summarized using descriptive statistics and will be presented by type of drug (WHO DRUG classification preferred term and Anatomical Therapeutic Chemical Classification [ATC] level 1) by treatment group.

8.4.2 Clinical Laboratory Tests

Hematology and chemistry data will be listed for each subject. Laboratory data will summarize only 10 mL dose groups by placebo or Ampion™. Values outside the normal laboratory reference range will be flagged as high or low on the listings. Depending on the size and scope of changes in laboratory data, summaries over time and/or changes from baseline over time may be provided. Similarly, depending on the size and scope of the changes, shift tables showing baseline to post-baseline categorization that are below, within, and above normal range may be provided for values recorded at week 6 and week 12.

8.4.3 Vital Signs

Vital signs will be listed for each subject. These will include temperature, respiration, pulse, and blood pressure. Summaries over time and changes from baseline will be provided.

8.4.4 Physical Examinations

Any new or abnormal findings will be recorded as adverse events. Status of each body system will be summarized at each visit.

8.5 Efficacy Analyses

All efficacy variables will be assessed at Baseline (Day 0), Week 2 (Day 14 ± 7), Week 4 (Day 28 ± 7), Week 6 (Day 42 ± 7), Week 8 (Day 56 ± 7), Week 10 (Day 70 ± 7) and Week 12 (Day 84 ± 7).

Differences between treatments will be presented using mean differences and 95% confidence intervals. Endpoints will be compared between treatment groups (Ampion™ or placebo, 4 mL or 10 mL, and between pooled placebo and Ampion™).

A subset of efficacy tables will be stratified by prior knee injection history and by Kellgren Lawrence grade.

Except where otherwise specified, missing data will not be estimated or carried forward in any of the descriptive analyses. No multiple comparison adjustment will be done for the secondary efficacy analyses. If the data clearly violate the distribution assumptions of the model, then transformations may be applied or rank-based methods used.

8.5.1 Primary Effectiveness Endpoint

Change in WOMAC pain score, from baseline to Week 12, will be analyzed by analysis of covariance (ANCOVA). The covariate will be the baseline WOMAC pain score. The residuals of the model will be assessed in terms of normalcy and heteroscedasticity, in order to ensure that the model used is appropriate. Least squares means and 95% confidence intervals will be presented by treatment arm.

The primary effectiveness endpoint will be analyzed using the ITT analysis population. In order to preserve the randomization, any missing or out of window values at 12 weeks will be imputed. The primary method of imputation will be by last observation carried forward (LOCF). To determine the possible affect of missing data on the primary analysis, the following sensitivity analyses will be performed. Second, a multiple imputation approach (SAS PROC MI, using the default method MCMC along with PROC MIANALYZE) will be used. Third, baseline values will be used to replace missing week 12 data (resulting in no change). Finally, a conservative imputation approach will be performed where the mean change for the placebo arm will be used to impute missing 12-week values in the Ampion™ arm; similarly, missing placebo values will be replaced by the mean Ampion™ arm decrease.

An analysis of site effect will be performed. If the main effect has a p-value of 0.15 or smaller, Site will be included in the model and summaries of the primary effectiveness endpoint will be produced by site as well as pooled over sites.

WOMAC Pain scores and change from baseline will be summarized by visit. Change from baseline will include a 95% CI. A test for the main effect for Ampion™ vs placebo will be included on the summary tables at weeks indicated as primary or secondary endpoints. ANOVA with adjustment for baseline and mL of injection will be included in the model.

8.5.2 Secondary Effectiveness Endpoints

Secondary effectiveness endpoints will be analyzed using the ITT and the PP analysis populations. Change from baseline WOMAC pain, stiffness and physical function, PGA, and WOMAC resting and moving pain subscales will be analyzed at weeks 2 through 12 (with the exception of the PGA where weeks 6-12 will be analyzed) using a mixed-effects repeated measures ANCOVA model (SAS PROC MIXED). The covariance structure will be modeled as first-order autoregressive [AR (1)]; subject will be designated a random effect. The covariate will be the baseline measure of the endpoint being analyzed. Treatment arm and Time (linear) will be included in the model. The interaction between treatment and time (linear) will be examined and removed from the

model if not significant. A quadratic trend for time and quadratic time by treatment interaction will be explored in the same manner. Tests for difference between treatment arms at each visit will be performed.

OMERACT-OARSI responder status will be analyzed by a general estimating equation model (PROC GENMOD) using a logistic link function and an AR (1) covariance structure for repeated measures. The baseline measures of WOMAC pain and baseline PGA will be included as covariates. Treatment arm and Time (linear) will be included in the model. The interaction between treatment and time (linear) will be examined and removed from the model if not significant. A quadratic trend for time and time by treatment interaction will be explored in the same manner as previously described.

Use of rescue analgesia will be compared by treatment arm. Fisher's exact tests will be performed at each visit to determine difference in use of rescue analgesia in the past 12 hours between placebo and Ampion™. A rank-sum test will be used to compare overall pill count use over the study between placebo and Ampion™.

Change in WORMS scales, focusing on MFT and LFT, will be evaluated by examining the paired difference between baseline and week 12 using 95% confidence intervals to test for no change for each treatment arm. Difference between placebo and Ampion™ will be evaluated by rank-based methods. Biomarkers will be summarized in a similar manner.

Continuous secondary endpoints and their change from baseline will be summarized by visit and treatment arm. Change from baseline will include a 95% CI. A main effect test for Ampion™ vs placebo will be included on the summary tables at weeks indicated as secondary endpoints. ANOVA with adjustment for baseline and mL of injection volume will be included in the model.

Responder status will be summarized at each visit for each treatment arm by number and percentage. Logistic regression, with adjustment for injection volume and baseline WOMAC pain and PGA, will be used to test for differences in responder status by placebo versus Ampion™ at each visit.

8.5.3 Exploratory Analysis

As WOMAC data are collected by two methods (in person at baseline, weeks 6 and 12; by telephone at weeks 2, 4, 8, and 10), an exploratory analysis will be performed to determine if data collection method has an impact on WOMAC pain scores. The model will include terms for time (linear and quadratic), treatment, flag for visit/telephone. The main effect for the flag will determine if pain scores

are lower or higher by one method over the other. An interaction between method and treatment will determine if the effect is consistent by treatment, and an interaction between time, treatment, and flag will examine differential time trends. The PP analysis population used, as only observed data are of interest in this analysis.

8.5.4 Multiplicity Adjustment

The secondary analyses are considered supportive to the primary analysis (i.e., not required to demonstrate efficacy of the test article); consequently, there is no requirement under ICH to adjust for multiplicity.

8.5.5 Post Hoc/Ad Hoc Analyses

Any analysis not described in this plan will be considered post-hoc and identified as such in the CSR.

9. CHANGES FROM PROTOCOL-SPECIFIED ANALYSES

Modifications to the planned statistical analyses should be minimized. None the less, the data obtained from the study may indicate that the planned analyses are inappropriate, that additional analyses need to be performed, or that the design of the study needs to be modified, due to factors such as the distribution of the data or imbalance in important covariates. The study report will provide a detailed explanation for any deviations from the planned analyses.

10. LIST OF PLANNED TABLES, FIGURES, AND LISTINGS

Tables are categorized and numbered in accordance with ICH E3 guidelines. Each table, figure and listing is presented by treatment arm. In addition to the 4 study arms, two columns will be added, one for combined placebo arms and the other for combined Ampion™ arms. Efficacy tables will be provided both for ITT and PP. Accountability tables will also include an overall column. Listings will be sorted by treatment, subject ID, and by visit, if multiple visits exist.

10.1 Tables

14.1 Accountability

14.1.1	Accountability (Analysis population: All Enrolled)
14.1.2	Enrollment by Site (Analysis population: All Enrolled)
14.1.3	Analysis Populations (Analysis population: All Enrolled)
14.1.4	Subject Disposition (All Screened)
14.1.5	Major Protocol Deviations (Analysis population: All Enrolled)
14.1.6.1	Demographics and Baseline Characteristics (Analysis population: ITT)
14.1.6.2	Demographics and Baseline Characteristics (Analysis population: PP)

14.2 Efficacy

14.2.1.1	Summary of WOMAC A Pain Subscale (Analysis population: ITT)
14.2.2.1	Summary of WOMAC B Stiffness Subscale (Analysis population: ITT)
14.2.3.1	Summary of WOMAC C Physical Function Subscale (Analysis population: ITT)
14.2.4.1	Summary of WOMAC A Pain with Movement Subscale (Analysis population: ITT)
14.2.5.1	Summary of WOMAC A Pain at Rest Subscale (Analysis population: ITT)
14.2.1.1.1	Summary of WOMAC A Pain Subscale (Analysis population: ITT with Prior Knee Injection)
14.2.2.1.1	Summary of WOMAC B Stiffness Subscale (Analysis population: ITT with Prior Knee Injection)
14.2.3.1.1	Summary of WOMAC C Physical Function Subscale (Analysis population: ITT with Prior Knee Injection)
14.2.4.1.1	Summary of WOMAC A Pain with Movement Subscale (Analysis population: ITT with Prior Knee Injection)
14.2.5.1.1	Summary of WOMAC A Pain at Rest Subscale (Analysis population: ITT with Prior Knee Injection)
14.2.1.2.1	Summary of WOMAC A Pain Subscale (Analysis population: ITT with no Prior Knee Injection)
14.2.2.2.1	Summary of WOMAC B Stiffness Subscale (Analysis population: ITT with no Prior Knee Injection)
14.2.3.2.1	Summary of WOMAC C Physical Function Subscale (Analysis population: ITT with no Prior Knee Injection)
14.2.4.2.1	Summary of WOMAC A Pain with Movement Subscale (Analysis population: ITT with no Prior Knee Injection)
14.2.5.2.1	Summary of WOMAC A Pain at Rest Subscale (Analysis population: ITT with no Prior Knee Injection)
14.2.1.3.1	Summary of WOMAC A Pain Subscale (Analysis population: ITT with Kellgren Lawrence Grade of 2)
14.2.2.3.1	Summary of WOMAC B Stiffness Subscale (Analysis population: ITT with Kellgren Lawrence Grade of 2)
14.2.3.3.1	Summary of WOMAC C Physical Function Subscale (Analysis population: ITT with Kellgren Lawrence Grade of 2)
14.2.4.3.1	Summary of WOMAC A Pain with Movement Subscale (Analysis population: ITT with Kellgren Lawrence Grade of 2)
14.2.5.3.1	Summary of WOMAC A Pain at Rest Subscale (Analysis population: ITT with Kellgren Lawrence Grade of 2)
14.2.1.4.1	Summary of WOMAC A Pain Subscale (Analysis population: ITT with Kellgren Lawrence Grade of 3)
14.2.2.4.1	Summary of WOMAC B Stiffness Subscale (Analysis population: ITT with Kellgren Lawrence Grade of 3)
14.2.3.4.1	Summary of WOMAC C Physical Function Subscale (Analysis population: ITT with Kellgren Lawrence Grade of 3)
14.2.4.4.1	Summary of WOMAC A Pain with Movement Subscale (Analysis population: ITT with Kellgren Lawrence Grade of 3)
14.2.5.4.1	Summary of WOMAC A Pain at Rest Subscale (Analysis population: ITT with Kellgren Lawrence Grade of 3)
14.2.1.5.1	Summary of WOMAC A Pain Subscale (Analysis population: ITT with Kellgren Lawrence Grade of 4 or 5)
14.2.2.5.1	Summary of WOMAC B Stiffness Subscale (Analysis population: ITT with Kellgren Lawrence Grade of 4 or 5)
14.2.3.5.1	Summary of WOMAC C Physical Function Subscale (Analysis population: ITT with Kellgren Lawrence Grade of 4 or 5)
14.2.4.5.1	Summary of WOMAC A Pain with Movement Subscale (Analysis population: ITT with

	Kellgren Lawrence Grade of 4 or 5)
14.2.5.5.1	Summary of WOMAC A Pain at Rest Subscale (Analysis population: ITT with Kellgren Lawrence Grade of 4 or 5)
14.2.1.2	Summary of WOMAC A Pain Subscale (Analysis population: PP)
14.2.2.2	Summary of WOMAC B Stiffness Subscale (Analysis population: PP)
14.2.3.2	Summary of WOMAC C Physical Function Subscale (Analysis population: PP)
14.2.4.2	Summary of WOMAC A Pain with Movement Subscale (Analysis population: PP)
14.2.5.2	Summary of WOMAC A Pain at Rest Subscale (Analysis population: PP)
14.2.1.1.2	Summary of WOMAC A Pain Subscale (Analysis population: PP with Prior Knee Injection)
14.2.2.1.2	Summary of WOMAC B Stiffness Subscale (Analysis population: PP with Prior Knee Injection)
14.2.3.1.2	Summary of WOMAC C Physical Function Subscale (Analysis population: PP with Prior Knee Injection)
14.2.4.1.2	Summary of WOMAC A Pain with Movement Subscale (Analysis population: PP with Prior Knee Injection)
14.2.5.1.2	Summary of WOMAC A Pain at Rest Subscale (Analysis population: PP with Prior Knee Injection)
14.2.1.2.2	Summary of WOMAC A Pain Subscale (Analysis population: PP with no Prior Knee Injection)
14.2.2.2.2	Summary of WOMAC B Stiffness Subscale (Analysis population: PP with no Prior Knee Injection)
14.2.3.2.2	Summary of WOMAC C Physical Function Subscale (Analysis population: PP with no Prior Knee Injection)
14.2.4.2.2	Summary of WOMAC A Pain with Movement Subscale (Analysis population: PP with no Prior Knee Injection)
14.2.5.2.2	Summary of WOMAC A Pain at Rest Subscale (Analysis population: PP with no Prior Knee Injection)
14.2.1.3.2	Summary of WOMAC A Pain Subscale (Analysis population: PP with Kellgren Lawrence Grade of 2)
14.2.2.3.2	Summary of WOMAC B Stiffness Subscale (Analysis population: PP with Kellgren Lawrence Grade of 2)
14.2.3.3.2	Summary of WOMAC C Physical Function Subscale (Analysis population: PP with Kellgren Lawrence Grade of 2)
14.2.4.3.2	Summary of WOMAC A Pain with Movement Subscale (Analysis population: PP with Kellgren Lawrence Grade of 2)
14.2.5.3.2	Summary of WOMAC A Pain at Rest Subscale (Analysis population: PP with Kellgren Lawrence Grade of 2)
14.2.1.4.2	Summary of WOMAC A Pain Subscale (Analysis population: PP with Kellgren Lawrence Grade of 3)
14.2.2.4.2	Summary of WOMAC B Stiffness Subscale (Analysis population: PP with Kellgren Lawrence Grade of 3)
14.2.3.4.2	Summary of WOMAC C Physical Function Subscale (Analysis population: PP with Kellgren Lawrence Grade of 3)
14.2.4.4.2	Summary of WOMAC A Pain with Movement Subscale (Analysis population: PP with Kellgren Lawrence Grade of 3)
14.2.5.4.2	Summary of WOMAC A Pain at Rest Subscale (Analysis population: PP with Kellgren Lawrence Grade of 3)
14.2.1.5.2	Summary of WOMAC A Pain Subscale (Analysis population: PP with Kellgren Lawrence Grade of 4 or 5)
14.2.2.5.2	Summary of WOMAC B Stiffness Subscale (Analysis population: PP with Kellgren Lawrence Grade of 4 or 5)
14.2.3.5.2	Summary of WOMAC C Physical Function Subscale (Analysis population: PP with Kellgren Lawrence Grade of 4 or 5)
14.2.4.5.2	Summary of WOMAC A Pain with Movement Subscale (Analysis population: PP with Kellgren Lawrence Grade of 4 or 5)
14.2.5.5.2	Summary of WOMAC A Pain at Rest Subscale (Analysis population: PP with Kellgren

	Lawrence Grade of 4 or 5)
14.2.7.1	Summary of WOMAC A Pain Subscale at Week 12 (Analysis population: ITT)
14.2.7.2	Summary of WOMAC A Pain Subscale at Week 12 (Analysis population: PP)
14.2.8.1	Primary WOMAC A Pain Subscale Analysis and Sensitivity Analyses at Week 12 (Analysis population: ITT)
14.2.9.1	Summary of PGA over Time (Analysis population: ITT)
14.2.9.2.1	Summary of PGA over Time (Analysis population: ITT with Prior Knee Injection)
14.2.9.3.1	Summary of PGA over Time (Analysis population: ITT with no Prior Knee Injection)
14.2.9.2	Summary of PGA over Time (Analysis population: PP)
14.2.9.2.2	Summary of PGA over Time (Analysis population: PP with Prior Knee Injection)
14.2.9.3.2	Summary of PGA over Time (Analysis population: PP with no Prior Knee Injection)
14.2.10.1	Summary of Rescue Analgesia Use Prior to Visit (Analysis population: ITT)
14.2.10.2	Summary of Rescue Analgesia Use Prior to Visit (Analysis population: PP)
14.2.10.3	Summary of Rescue Analgesia Use (Analysis population: ITT)
14.2.10.4	Summary of Rescue Analgesia Use (Analysis population: PP)
14.2.11.1	Summary of OMERACT-OARSI Response over Time (Analysis population: ITT)
14.2.11.1.1	Summary of OMERACT-OARSI Response over Time (Analysis population: ITT with Prior Knee Injection)
14.2.11.1.2	Summary of OMERACT-OARSI Response over Time (Analysis population: ITT with no Prior Knee Injection)
14.2.11.2	Summary of OMERACT-OARSI Response over Time (Analysis population: PP)
14.2.11.2.1	Summary of OMERACT-OARSI Response over Time (Analysis population: PP with Prior Knee Injection)
14.2.11.2.2	Summary of OMERACT-OARSI Response over Time (Analysis population: PP with no Prior Knee Injection)

14.3 Safety

14.3.1	Overall Summary of Treatment-Emergent Adverse Events (Analysis population: Safety)
14.3.2	Incidence of Treatment-Emergent Adverse Events by System Organ Class and Preferred Term (Analysis population: Safety)
14.3.3	Incidence of Treatment-Emergent Related Adverse Events by System Organ Class and Preferred Term (Analysis population: Safety)
14.3.4	Incidence of Treatment-Emergent Serious Adverse Events by System Organ Class and Preferred Term (Analysis population: Safety)
14.3.5	Incidence of Treatment-Emergent Adverse Events by Preferred Term in Descending Order of Frequency (Analysis population: Safety)
14.3.6	Incidence of Treatment-Emergent Related Adverse Events by Preferred Term in Descending Order of Frequency (Analysis population: Safety)
14.3.7.1	Summary of Laboratory Value and Change from Baseline over Time for Hemoglobin (g/L) (Analysis population: Safety)
14.3.7.2	Summary of Laboratory Value and Change from Baseline over Time for Red Cell Count (x 10^12/L) (Analysis population: Safety)
14.3.7.3	Summary of Laboratory Value and Change from Baseline over Time for Haematocrit (%) (Analysis population: Safety)
14.3.7.4	Summary of Laboratory Value and Change from Baseline over Time for Mean Cell Volume (fl) (Analysis population: Safety)
14.3.7.5	Summary of Laboratory Value and Change from Baseline over Time for Mean Cell Hemoglobin (pg) (Analysis population: Safety)
14.3.7.6	Summary of Laboratory Value and Change from Baseline over Time for Mean Cell Hemoglobin Conc. (g/L) (Analysis population: Safety)
14.3.7.7	Summary of Laboratory Value and Change from Baseline over Time for Platelets (x 10^9/L) (Analysis population: Safety)
14.3.7.8	Summary of Laboratory Value and Change from Baseline over Time for White Cell Counts (x 10^9/L) (Analysis population: Safety)

14.3.7.9	Summary of Laboratory Value and Change from Baseline over Time for Neutrophils (x 10^9/L) (Analysis population: Safety)
14.3.7.10	Summary of Laboratory Value and Change from Baseline over Time for Lymphocytes (x 10^9/L) (Analysis population: Safety)
14.3.7.11	Summary of Laboratory Value and Change from Baseline over Time for Monocytes (x 10^9/L) (Analysis population: Safety)
14.3.7.12	Summary of Laboratory Value and Change from Baseline over Time for Eosinophils (x 10^9/L) (Analysis population: Safety)
14.3.7.13	Summary of Laboratory Value and Change from Baseline over Time for Basophils (x 10^9/L) (Analysis population: Safety)
14.3.7.14	Summary of Laboratory Value and Change from Baseline over Time for Sodium (mmol/L) (Analysis population: Safety)
14.3.7.15	Summary of Laboratory Value and Change from Baseline over Time for Potassium (mmol/L) (Analysis population: Safety)
14.3.7.16	Summary of Laboratory Value and Change from Baseline over Time for Chloride (mmol/L) (Analysis population: Safety)
14.3.7.17	Summary of Laboratory Value and Change from Baseline over Time for Bicarbonate (mmol/L) (Analysis population: Safety)
14.3.7.18	Summary of Laboratory Value and Change from Baseline over Time for Urea (mmol/L) (Analysis population: Safety)
14.3.7.19	Summary of Laboratory Value and Change from Baseline over Time for Creatinine (umol/L) (Analysis population: Safety)
14.3.7.20	Summary of Laboratory Value and Change from Baseline over Time for Urate (mmol/L) (Analysis population: Safety)
14.3.7.21	Summary of Laboratory Value and Change from Baseline over Time for Glucose (mmol/L) (Analysis population: Safety)
14.3.7.22	Summary of Laboratory Value and Change from Baseline over Time for Cholesterol (mmol/L) (Analysis population: Safety)
14.3.7.23	Summary of Laboratory Value and Change from Baseline over Time for Lactate Dehydrogenase (U/L) (Analysis population: Safety)
14.3.7.24	Summary of Laboratory Value and Change from Baseline over Time for Total Calcium (mmol/L) (Analysis population: Safety)
14.3.7.25	Summary of Laboratory Value and Change from Baseline over Time for Phosphate (mmol/L) (Analysis population: Safety)
14.3.7.26	Summary of Laboratory Value and Change from Baseline over Time for Protein (g/L) (Analysis population: Safety)
14.3.7.27	Summary of Laboratory Value and Change from Baseline over Time for Albumin (g/L) (Analysis population: Safety)
14.3.7.28	Summary of Laboratory Value and Change from Baseline over Time for Globulins (g/L) (Analysis population: Safety)
14.3.7.29	Summary of Laboratory Value and Change from Baseline over Time for Creatinine Kinase (U/L) (Analysis population: Safety)
14.3.7.30	Summary of Laboratory Value and Change from Baseline over Time for Total Bilirubin (umol/L) (Analysis population: Safety)
14.3.7.31	Summary of Laboratory Value and Change from Baseline over Time for Conjugated Bilirubin (umol/L) (Analysis population: Safety)
14.3.7.32	Summary of Laboratory Value and Change from Baseline over Time for Alanine Aminotransferase (U/L) (Analysis population: Safety)
14.3.7.33	Summary of Laboratory Value and Change from Baseline over Time for Aspartate Aminotransferase (U/L) (Analysis population: Safety)
14.3.7.34	Summary of Laboratory Value and Change from Baseline over Time for Gamma Glutamyltransferase (U/L) (Analysis population: Safety)
14.3.7.35	Summary of Laboratory Value and Change from Baseline over Time for Alkaline Phosphatase (U/L) (Analysis population: Safety)
14.3.7.36	Summary of Laboratory Value and Change from Baseline over Time for Triglycerides (mmol/L) (Analysis population: Safety)

14.3.8.1	Shifts in Reference Range for Hemoglobin (g/L) (Analysis population: Safety)
14.3.8.2	Shifts in Reference Range for Red Cell Count (x 10^12/L) (Analysis population: Safety)
14.3.8.3	Shifts in Reference Range for Haematocrit (%) (Analysis population: Safety)
14.3.8.4	Shifts in Reference Range for Mean Cell Volume (fl) (Analysis population: Safety)
14.3.8.5	Shifts in Reference Range for Mean Cell Hemoglobin (pg) (Analysis population: Safety)
14.3.8.6	Shifts in Reference Range for Mean Cell Hemoglobin Conc. (g/L) (Analysis population: Safety)
14.3.8.7	Shifts in Reference Range for Platelets (x 10^9/L) (Analysis population: Safety)
14.3.8.8	Shifts in Reference Range for White Cell Counts (x 10^9/L) (Analysis population: Safety)
14.3.8.9	Shifts in Reference Range for Neutrophils (x 10^9/L) (Analysis population: Safety)
14.3.8.10	Shifts in Reference Range for Lymphocytes (x 10^9/L) (Analysis population: Safety)
14.3.8.11	Shifts in Reference Range for Monocytes (x 10^9/L) (Analysis population: Safety)
14.3.8.12	Shifts in Reference Range for Eosinophils (x 10^9/L) (Analysis population: Safety)
14.3.8.13	Shifts in Reference Range for Basophils (x 10^9/L) (Analysis population: Safety)
14.3.8.14	Shifts in Reference Range for Sodium (mmol/L) (Analysis population: Safety)
14.3.8.15	Shifts in Reference Range for Potassium (mmol/L) (Analysis population: Safety)
14.3.8.16	Shifts in Reference Range for Chloride (mmol/L) (Analysis population: Safety)
14.3.8.17	Shifts in Reference Range for Bicarbonate (mmol/L) (Analysis population: Safety)
14.3.8.18	Shifts in Reference Range for Urea (mmol/L) (Analysis population: Safety)
14.3.8.19	Shifts in Reference Range for Creatinine (umol/L) (Analysis population: Safety)
14.3.8.20	Shifts in Reference Range for Urate (mmol/L) (Analysis population: Safety)
14.3.8.21	Shifts in Reference Range for Glucose (mmol/L) (Analysis population: Safety)
14.3.8.22	Shifts in Reference Range for Cholesterol (mmol/L) (Analysis population: Safety)
14.3.8.23	Shifts in Reference Range for Lactate Dehydrogenase (U/L) (Analysis population: Safety)
14.3.8.24	Shifts in Reference Range for Total Calcium (mmol/L) (Analysis population: Safety)
14.3.8.25	Shifts in Reference Range for Phosphate (mmol/L) (Analysis population: Safety)
14.3.8.26	Shifts in Reference Range for Protein (g/L) (Analysis population: Safety)
14.3.8.27	Shifts in Reference Range for Albumin (g/L) (Analysis population: Safety)
14.3.8.28	Shifts in Reference Range for Globulins (g/L) (Analysis population: Safety)
14.3.8.29	Shifts in Reference Range for Creatinine Kinase (U/L) (Analysis population: Safety)
14.3.8.30	Shifts in Reference Range for Total Bilirubin (umol/L) (Analysis population: Safety)
14.3.8.31	Shifts in Reference Range for Conjugated Bilirubin (umol/L) (Analysis population: Safety)
14.3.8.32	Shifts in Reference Range for Alanine Aminotransferase (U/L) (Analysis population: Safety)
14.3.8.33	Shifts in Reference Range for Aspartate Aminotransferase (U/L) (Analysis population: Safety)
14.3.8.34	Shifts in Reference Range for Gamma Glutamyltransferase (U/L) (Analysis population: Safety)
14.3.8.35	Shifts in Reference Range for Alkaline Phosphatase (U/L) (Analysis population: Safety)
14.3.8.36	Shifts in Reference Range for Triglycerides (mmol/L) (Analysis population: Safety)
14.3.9.1	Summary of Vital Signs and Change from Baseline over Time for Pulse (bpm) (Analysis population: Safety)
14.3.9.2	Summary of Vital Signs and Change from Baseline over Time for Temperature (F) (Analysis population: Safety)
14.3.9.3	Summary of Vital Signs and Change from Baseline over Time for Systolic BP (mmHg) (Analysis population: Safety)
14.3.9.4	Summary of Vital Signs and Change from Baseline over Time for Diastolic BP (mmHg) (Analysis population: Safety)
14.3.10.1	Concomitant Medication Use by ATC Level 1 and WHO Preferred Term (Analysis population: Safety)
14.3.10.2	Medication Started on Study by ATC Level 1 and WHO Preferred Term (Analysis population: Safety)
14.3.10.3	Concomitant Medications by WHO Preferred Term in Descending Order of Use (Analysis population: Safety)
14.3.10.4	Medication Started on Study by WHO Preferred Term in Descending Order of Use (Analysis population: Safety)

Adverse Events

10.2 Listings

Subject Accountability

1. Randomization List (including, subject ID, randomization number, randomized treatment and treatment administered, and date of treatment)
2. Inclusion and Exclusion Criteria
3. Protocol Deviations
4. Subjects Withdrawing from the Study Prematurely (date and reason)
5. Analysis Populations with Reason for Exclusion (if populations differ)

Demographics and Baseline Characteristics

6. Demographics and Baseline Characteristics
[age, sex, race, ethnicity, weight, height, BMI, and baseline knee evaluation, Kellgren Lawrence grade, prior injection history, baseline WOMAC and PGA scores.]
7. Medical History
8. Baseline Medication Use

Efficacy

9. WOMAC A, B, and C Scores (includes all subscales)
10. PGA Scores
11. OMERACT-OARSI Response
12. Rescue Analgesia
13. WORMS Subscores (focus on MFT and LFT)
14. Biomarker Values

Safety

15. All Adverse Events [with indication of TEAE]
16. Physical Exams
17. Hematology Data [with flagging of values outside of normal range]
18. Chemistry Data [with flagging of values outside of normal range]

19. Vital Signs Data
[systolic and diastolic blood pressure, pulse rate, respiration, and temperature]

10.3 Figures

Note that all figures, unless otherwise stated, will be line plots showing mean ± SEM at each visit for each treatment arm.

1. WOMAC A Pain Subscale at Week 12 [Boxplot]
2. Summary of WOMAC A Pain Subscale over Time
3. Summary of WOMAC B Stiffness Subscale over Time
4. Summary of WOMAC C Physical Function Subscale over Time
5. Summary of PGA over Time
6. Summary of OMERACT-OARSI Response over Time [stacked histogram]
7. Summary of WOMAC A Pain with Movement Subscale over Time
8. Summary of WOMAC A Pain at Rest Subscale over Time
9. Summary of Rescue Analgesia Used [stacked histogram]

11. LITERATURE CITATIONS / REFERENCES

SAS Institute Inc. SAS Language: version 8 first edition. SAS Institute, Inc, Cary, NC, USA, 1990.

R Core Team (2012). R: A language and environment for statistical computing. R Foundation for Statistical Computing, Vienna, Austria. ISBN 3-900051-07-0, URL: http://www.R-project.org/.

12. HANDLING OF MISSING OR INCOMPLETE DATES FOR ADVERSE EVENTS AND CONCOMITANT MEDICATIONS

12.1 Imputation Rules for Partial or Missing Stop Dates

If the month and year are present, impute the last day of the month. If only the year is present, impute December 31 of that year. If the stop date is entirely missing, assume the event or medication is ongoing. If a partial or complete stop date is present and the 'ongoing' or 'continuing' box is checked, then it will be assumed that the AE or concomitant medication stopped and the stop date will be imputed, if partial.

<table>
<tr><th></th><th colspan="8">Stop Date</th></tr>
<tr><th></th><th></th><th colspan="2">Complete: yyyymmdd</th><th colspan="2">Partial: yyyymm</th><th colspan="2">Partial: yyyy</th><th rowspan="2">Missing</th></tr>
<tr><th>Start Date</th><th></th><th><1st Dose</th><th>≥1st Dose</th><th><1st Dose yyyymm</th><th>≥1st Dose yyyymm</th><th><1st Dose yyyy</th><th>≥1st Dose yyyy</th></tr>
<tr><td rowspan="2">Partial: yyyymm</td><td>=1st Dose yyyymm</td><td rowspan="2">2</td><td>1</td><td rowspan="2">2</td><td>1</td><td>N/A</td><td>1</td><td>1</td></tr>
<tr><td>≠ 1st Dose yyyymm</td><td>2</td><td>2</td><td>2</td><td>2</td><td>2</td></tr>
<tr><td rowspan="2">Partial: yyyy</td><td>=1st Dose yyyy</td><td rowspan="2">3</td><td>1</td><td rowspan="2">3</td><td>1</td><td>N/A</td><td>1</td><td>1</td></tr>
<tr><td>≠ 1st Dose yyyy</td><td>3</td><td>3</td><td>3</td><td>3</td><td>3</td></tr>
<tr><td colspan="2">Missing</td><td>4</td><td>1</td><td>4</td><td>1</td><td>4</td><td>1</td><td>1</td></tr>
</table>

1 = Impute the date of first dose
2 = Impute the first of the month
3 = Impute January 1 of the year
4 = Impute January 1 of the stop year

Note: For subjects who were never treated (first dose date is missing), partial start dates will be set to the first day of the partial month.

Note: If the start date imputation leads to a start date that is after the stop date, then do not impute the start date.

13. SCHEDULE OF ASSESSMENTS AND PROCEDURES

	Screening	Baseline Random-ization Treatment	Post-treatment check (telephone contact)	Week 2 (telephone contact)	Week 4 (telephone contact)	Week 6	Week 8 (telephone contact)	Week 10 (telephone contact)	Week 12 Final Visit	Early Termination
Visit # Day #	1 Day-28 to 0	2 Day 0	3 Day 1	4 Day 14 ± 7	5 Day 28 ± 7	6 Day 42 ± 7	7 Day 56 ± 7	8 Day 70 ± 7	9 Day 84 ± 7	
Informed Consent	X									
Inclusion/exclusion criteria	X									
Medical history/prior medications	X									
Conconcomitant medications	X	X	X	X	X	X	X	X	X	X
Physical examination	X	X				X			X	X
Vital Signs	X	X				X			X	X
Randomization		X								
WOMAC	X	X		X	X	X	X	X	X	X
Patient's global assessment (PGA)	X	X		X	X	X	X	X	X	X
X-ray[1]	X									
MRI (WORMS)[2]		X							X	
Knee aspiration[3]		X				X			X	
Clinical laboratory tests[4]		X				X			X	X
Treatment with study drug		X								
Rescue medication		X								
Review Rescue medication			X	X	X	X	X	X	X	X
Adverse Events		X	X	X	X	X	X	X	X	X

Visits are in clinic except for Days 2 and Weeks 2, 4, 8 and 10 when patients will be contacted by telephone

1 X-ray may be acquired at Screening to satisfy inclusion criteria, "Index knee must be symptomatic for greater than 6 months with a clinical diagnosis of OA and supported by radiological evidence (Kellgren Lawrence Grade II to IV) that is not older than 6 months prior to the date of screening".

2 Only applicable for patients randomized into the subset of 20 patients in the 10mL injection study arms at one site.

3 Only applicable for patients randomized into the subset of 20 patients in the 10mL injection study arms at one site.

4 Only applicable for patients randomized into the subset of 20 patients in the 10mL injection study arms at one site.
