# Supplementary material for: A Randomized Clinical Trial to Evaluate Two Doses of an Intra-Articular Injection of LMWF-5A in Adults with Pain Due to Osteoarthritis of the Knee
Source: PLoS One. 2014 Feb 3;9(2):e87910. doi: 10.1371/journal.pone.0087910 (PMC3912151; doi:10.1371/journal.pone.0087910)
Supplement: Issued Patents S1 — Issued Patents for “AMPION”, as of December 5, 2013. (DOCX) [file pone.0087910.s004.docx]

Issued Patents for "AMPION", as of December 5, 2013

| **Reference #** | **Title** | **Country** | **Serial #** | **Filed Date** | **Patent #** | **Issue Date** | **Status** | **Expiration** |
| --- | --- | --- | --- | --- | --- | --- | --- | --- |
| 6134-36-PGB | Method of Synthesizing Diketopiperazines | GB | 0301542.7 | 8/2/2001 | GB2382346 | 8/11/2004 | Issued | 8/2/2021 |
| 6134-36 | Method of Synthesizing Diketopiperazines | US | 09/922,604 | 8/2/2001 | 6,967,202 | 11/22/2005 | Issued | 7/21/2022 |
| 6134-4-PEPAT | Method of Using Diketopiperazines and Composition Containing Them | AT | 01957581.0 | 8/2/2001 | 1311269 | 2/29/2012 | Issued | 8/2/2021 |
| 6134-4-PAU | Method of Using Diketopiperazines and Composition Containing Them | AU | 2001279313 | 8/2/2001 | 2001279313 | 6/14/2007 | Issued | 8/2/2021 |
| 6134-4-PEPBE | Method of Using Diketopiperazines and Composition Containing Them | BE | 01957581.0 | 8/2/2001 | 1311269 | 2/29/2012 | Issued | 8/2/2021 |
| 6134-4-PCA | Method of Using Diketopiperazines and Composition Containing Them | CA | 2,417,960 | 8/2/2001 | 2,417,960 | 7/10/2012 | Issued | 8/2/2021 |
| 6134-4-PEPCH | Method of Using Diketopiperazines and Composition Containing Them | CH | 01957581.0 | 8/2/2001 | 1311269 | 2/29/2012 | Issued | 8/2/2021 |
| 6134-4-PCN | Method of Using Diketopiperazines and Composition Containing Them | CN | 01815837.4 | 8/2/2001 | ZL01815837.4 | 10/28/2009 | Issued | 8/2/2021 |
| 6134-4-PCN-DIV | Method of Using Diketopiperazines and Composition Containing Them | CN | 200910145682.4 | 8/2/2001 | ZL200910145682.4 | 5/18/2011 | Issued | 8/2/2021 |
| 6134-4-PEPCY | Method of Using Diketopiperazines and Composition Containing Them | CY | 01957581.0 | 8/2/2001 | 1311269 | 2/29/2012 | Issued | 8/2/2021 |
| 6134-4-PEPDE | Method of Using Diketopiperazines and Composition Containing Them | DE | 01957581.0 | 8/2/2001 | 60146190.8 | 2/29/2012 | Issued | 8/2/2021 |
| 6134-4-PEPDK | Method of Using Diketopiperazines and Composition Containing Them | DK | 01957581.0 | 8/2/2001 | 1311269 | 2/29/2012 | Issued | 8/2/2021 |
| 6134-4-PEP | Method of Using Diketopiperazines and Composition Containing Them | EP | 01957581.0 | 8/2/2001 | 1311269 | 2/29/2012 | Issued | 8/2/2021 |
| 6134-4-PEPES | Method of Using Diketopiperazines and Composition Containing Them | ES | 01957581.0 | 8/2/2001 | 1311269 | 2/29/2012 | Issued | 8/2/2021 |
| 6134-4-PEPFI | Method of Using Diketopiperazines and Composition Containing Them | FI | 01957581.0 | 8/2/2001 | 1311269 | 2/29/2012 | Issued | 8/2/2021 |
| 6134-4-PEPFR | Method of Using Diketopiperazines and Composition Containing Them | FR | 01957581.0 | 8/2/2001 | 1311269 | 2/29/2012 | Issued | 8/2/2021 |
| 6134-4-PEPGB | Method of Using Diketopiperazines and Composition Containing Them | GB | 01957581.0 | 8/2/2001 | 1311269 | 2/29/2012 | Issued | 8/2/2021 |
| 6134-4-PEPGR | Method of Using Diketopiperazines and Composition Containing Them | GR | 01957581.0 | 8/2/2001 | 1311269 | 2/29/2012 | Issued | 8/2/2021 |
| 6134-4-PCNHK-DIV | Method of Using Diketopiperazines and Composition Containing Them | HK | 10103675.4 | 8/2/2001 | 1135333 | 2/3/2012 | Issued | 8/2/2021 |
| 6134-4-PEPIE | Method of Using Diketopiperazines and Composition Containing Them | IE | 01957581.0 | 8/2/2001 | 1311269 | 2/29/2012 | Issued | 8/2/2021 |
| 6134-4-PEPIT | Method of Using Diketopiperazines and Composition Containing Them | IT | 01957581.0 | 8/2/2001 | 1311269 | 2/29/2012 | Issued | 8/2/2021 |
| 6134-4-PJP | Method of Using Diketopeiperazines and Composition Containing Them | JP | 2002-517014 | 8/2/2001 | 5048201 | 7/27/2012 | Issued | 8/2/2021 |
| 6134-4-PEPLU | Method of Using Diketopiperazines and Composition Containing Them | LU | 01957581.0 | 8/2/2001 | 1311269 | 2/29/2012 | Issued | 8/2/2021 |
| 6134-4-PEPMC | Method of Using Diketopiperazines and Composition Containing Them | MC | 01957581.0 | 8/2/2001 | 1311269 | 2/29/2012 | Issued | 8/2/2021 |
| 6134-4-PEPNL | Method of Using Diketopiperazines and Composition Containing Them | NL | 01957581.0 | 8/2/2001 | 1311269 | 2/29/2012 | Issued | 8/2/2021 |
| 6134-4-PEPPT | Method of Using Diketopiperazines and Composition Containing Them | PT | 01957581.0 | 8/2/2001 | 1311269 | 2/29/2012 | Issued | 8/2/2021 |
| 6134-4-PEPSE | Method of Using Diketopiperazines and Composition Containing Them | SE | 01957581.0 | 8/2/2001 | 1311269 | 2/29/2012 | Issued | 8/2/2021 |
| 6134-4-PEPTR | Method of Using Diketopiperazines and Composition Containing Them | TR | 01957581.0 | 8/2/2001 | 1311269 | 2/29/2012 | Issued | 8/2/2021 |
| 6134-4-1 | Method of Using Diketopiperazines and Composition Containing Them | US | 10/397,964 | 3/25/2003 | 8,455,517 | 6/4/2013 | Issued | 8/2/2021 |
| 6134-4-1-1 | Method of Using Diketopiperazines and Composition Containing Them | US | 13/248,422 | 9/29/2011 | 8,440,696 | 5/14/2013 | Issued | 8/2/2021 |
| 6134-4-1-4 | Method of Using Diketopiperazines and Composition Containing Them | US | 13/338,441 | 12/28/2011 | 8,268,830 | 9/18/2012 | Issued | 8/2/2021 |
| 6134-4 | Method of Using Diketopiperazines and Composition Containing Them | US | 09/922,234 | 8/2/2001 | 6,555,543 | 4/29/2003 | Issued | 8/2/2021 |
| 6134-4-PZA | Method of Using Diketopeiperazines and Composition Containing Them | ZA | 2003/0934 | 8/2/2001 | 2003/0934 | 8/25/2004 | Issued | 8/2/2021 |
| 6134-84-PAU | Treatment of T-Cell Mediated Diseases | AU | 2004241101 | 5/14/2004 | 2004241101 | 8/26/2010 | Issued | 5/14/2024 |
| 6134-84-PIN | Treatment of T-Cell Mediated Diseases | IN | 5484/DELNP/2005 | 5/14/2004 | 233,807 | 4/8/2009 | Issued | 5/14/2024 |
| 6134-84-PNZ | Treatment of T-Cell Mediated Diseases | NZ | 542886 | 5/14/2004 | 542886 | 5/14/2004 | Issued | 5/14/2024 |
| 6134-84-PNZ-DIV | Treatment of T-Cell Mediated Diseases | NZ | 576931 | 5/14/2004 | 576931 | 4/5/2011 | Issued | 5/14/2024 |
| 6134-84-PNZ-DIV-2 | Treatment of T-Cell Mediated Diseases | NZ | 586516 | 5/14/2004 | 586516 | 4/2/2012 | Issued | 5/14/2024 |
| 6134-84-PSG | Treatment of T-Cell Mediated Diseases | SG | 200506561-0 | 5/14/2004 | 116214 | 2/29/2008 | Issued | 5/14/2024 |
| 6134-84 | Treatment of T-Cell Mediated Diseases | US | 10/846,482 | 5/14/2004 | 7,732,403 | 6/8/2010 | Issued | 5/14/2024 |
| 6134-84-DIV-2 | Treatment of T-Cell Mediated Diseases | US | 12/707,958 | 2/18/2010 | 8,324,167 | 12/4/2012 | Issued | 10/7/2024 |
| 6134-84-DIV2-1 | Treatment of T-Cell Mediated Diseases | US | 13/247,782 | 9/28/2011 | 8,183,209 | 5/22/2012 | Issued | 5/14/2024 |
| 6134-84-CON-1-2 | Treatment of T-Cell Mediated Diseases | US | 13/676,966 | 11/14/2012 | 8,551,953 | 10/8/2013 | Issued | 5/14/2024 |
| 6134-84-CON-1-3 | Treatment of T-Cell Mediated Diseases | US | 13/676,984 | 11/14/2012 | 8,513,196 | 8/20/2013 | Issued | 5/14/2024 |
| 6134-84-PZA | Treatment of T-Cell Mediated Diseases | ZA | 2005/09184 | 5/14/2004 | 2005/09184 | 4/25/2007 | Issued | 5/14/2024 |
